# Targeting the insulin-like growth factor receptor and Src signaling network for the treatment of non-small cell lung cancer

**DOI:** 10.1186/s12943-015-0392-3

**Published:** 2015-06-04

**Authors:** Hye-Young Min, Hye Jeong Yun, Ji-Sun Lee, Hyo-Jong Lee, Jaebeom Cho, Hyun-Ji Jang, Shin-Hyung Park, Diane Liu, Seung-Hyun Oh, J. Jack Lee, Ignacio I. Wistuba, Ho-Young Lee

**Affiliations:** College of Pharmacy and Research Institute of Pharmaceutical Sciences, Seoul National University, Seoul, 151-742 Republic of Korea; College of Pharmacy, Inje University, Gimhae, Gyungnam 621-749 Republic of Korea; College of Pharmacy, Gachon University, Incheon, 406-840 Republic of Korea; Department of Biostatistics, The University of Texas M. D. Anderson Cancer Cener, Houston, TX USA; Department of Thoracic/Head & Neck Medical Oncology, The University of Texas M. D. Anderson Cancer Cener, Houston, TX USA; Department of Pathology, The University of Texas M. D. Anderson Cancer Cener, Houston, TX USA

**Keywords:** Insulin-like growth factor receptor, Src, Linsitinib, Lung cancer

## Abstract

**Background:**

Therapeutic interventions in the insulin-like growth factor receptor (IGF-1R) pathway were expected to provide clinical benefits; however, IGF-1R tyrosine kinase inhibitors (TKIs) have shown limited antitumor efficacy, and the mechanisms conveying resistance to these agents remain elusive.

**Methods:**

The expression and activation of the IGF-1R and Src were assessed *via* the analysis of a publicly available dataset, as well as immunohistochemistry, Western blotting, RT-PCR, and *in vitro* kinase assays. The efficacy of IGF-1R TKIs alone or in combination with Src inhibitors was analyzed using MTT assays, colony formation assays, flow cytometric analysis, and xenograft tumor models.

**Results:**

The co-activation of IGF-1R and Src was observed in multiple human NSCLC cell lines as well as in a tissue microarray (n = 353). The IGF-1R and Src proteins mutually phosphorylate on their autophosphorylation sites. In high-pSrc-expressing NSCLC cells, linsitinib treatment initially inactivated the IGF-1R pathway but led a Src-dependent reactivation of downstream effectors. In low-pSrc-expressing NSCLC cells, linsitinib treatment decreased the turnover of the IGF-1R and Src proteins, ultimately amplifying the reciprocal co-activation of IGF-1R and Src. Co-targeting IGF-1R and Src significantly suppressed the proliferation and tumor growth of both high-pSrc-expressing and low-pSrc-expressing NSCLC cells *in vitro* and *in vivo* and the growth of patient-derived tissues *in vivo*.

**Conclusions:**

Reciprocal activation between Src and IGF-1R occurs in NSCLC. Src causes IGF-1R TKI resistance by acting as a key downstream modulator of the cross-talk between multiple membrane receptors. Targeting Src is a clinically applicable strategy to overcome resistance to IGF-1R TKIs.

**Electronic supplementary material:**

The online version of this article (doi:10.1186/s12943-015-0392-3) contains supplementary material, which is available to authorized users.

## Background

Non-small cell lung cancer (NSCLC) is one of the leading causes of cancer-related deaths worldwide [1], and the 5-year survival rate for patients with advanced NSCLC remains less than 20 % [2]. Although a small subset of patients with specific genetic and epigenetic abnormalities has shown clinical response to specific targeted therapies [3], most patients with NSCLCs are insensitive to chemotherapy and radiation^1^. Moreover, acquired resistance to the therapies eventually emerges in the initially sensitive patients after continuous treatment [4]. As activation of the survival potential seem to provide cancer cells resistance to anticancer therapies, it is likely that effective anticancer therapies must occur in parallel with blockade of key survival pathways.

The insulin-like growth factor receptor (IGF-1R) signaling pathway plays a critical role in cancer cell survival, causing resistance to numerous anticancer drugs [5–7]. Therefore, effective regimens to inactivate the IGF-1R pathway may sensitize cancer cells to anticancer therapies and provide clinical benefits to cancer patients. However, patients who show a promising initial response to anti-IGF-1R monoclonal antibodies (mAbs) appear to rapidly acquire resistance to these mAbs [8–10]. Similarly, therapeutic efficacy of IGF-1R TKIs has been modest in a variety of human cancers, including NSCLC^2^ thus, there is an urgent need to understand the signaling pathways that confer inherent and/or acquired resistance to anti-IGF-1R drugs and to develop new strategies to overcome this resistance.

The canonical activation of IGF-1R results from the binding of ligands (IGF1 and IGF2) to IGF-IR, insulin receptor (IR) (with a lower affinity than insulin), and hybrid receptors of IGF-1R/IR [6, 11], leading to the autophosphorylation of tyrosine residues 1131, 1135, and 1136 in the activation loop of the IGF-1R β-chain (the corresponding residues in the human IR are 1158, 1162, and 1163, respectively). Previous reports have suggested that Src, a non-receptor protein tyrosine kinase, can directly phosphorylate IGF-1R [12, 13]. Src also plays an important role in cancer cell survival and resistance to targeted anticancer therapies by acting as a common signaling facilitator that is activated by a myriad of redundant signaling pathways [12, 14]. The elevated expression and/or activation of Src and multiple membrane-associated receptors, such as IGF-1R, IR integrins, HER2/neu, and EGFR (all of which trigger Src activation), have been reported in NSCLCs [14–17]. Hence, it is likely that Src acts as a downstream node that links signalings among several collateral membrane associated receptors.

Here, we demonstrate high levels of Src and IGF-1R co-activation though mutual phosphorylation in the majority of NSCLC. We show that Src kinase activity plays a key role in *de novo* resistance to IGF-1R TKIs in NSCLC cells. NSCLC cells with high Src kinase activity can be independent from IGF-1R activation. Moreover, treatment of NSCLC cells with low Src kinase activity with an IGF-1R TKI enhances the reciprocal Src and IGF-1R activation *via* stabilization of IGF-1R and Src proteins. Finally, we show that Src antagonism universally sensitizes NSCLC cells to IGF-1R TKIs *in vitro* and *in vivo*. These results suggest that a combined treatment with IGF-1R and Src inhibitors is a useful and clinically applicable therapeutic strategy for NSCLC.

## Results

### Co-activation of IGF-1R and Src in human NSCLC

**Fig. 1:**
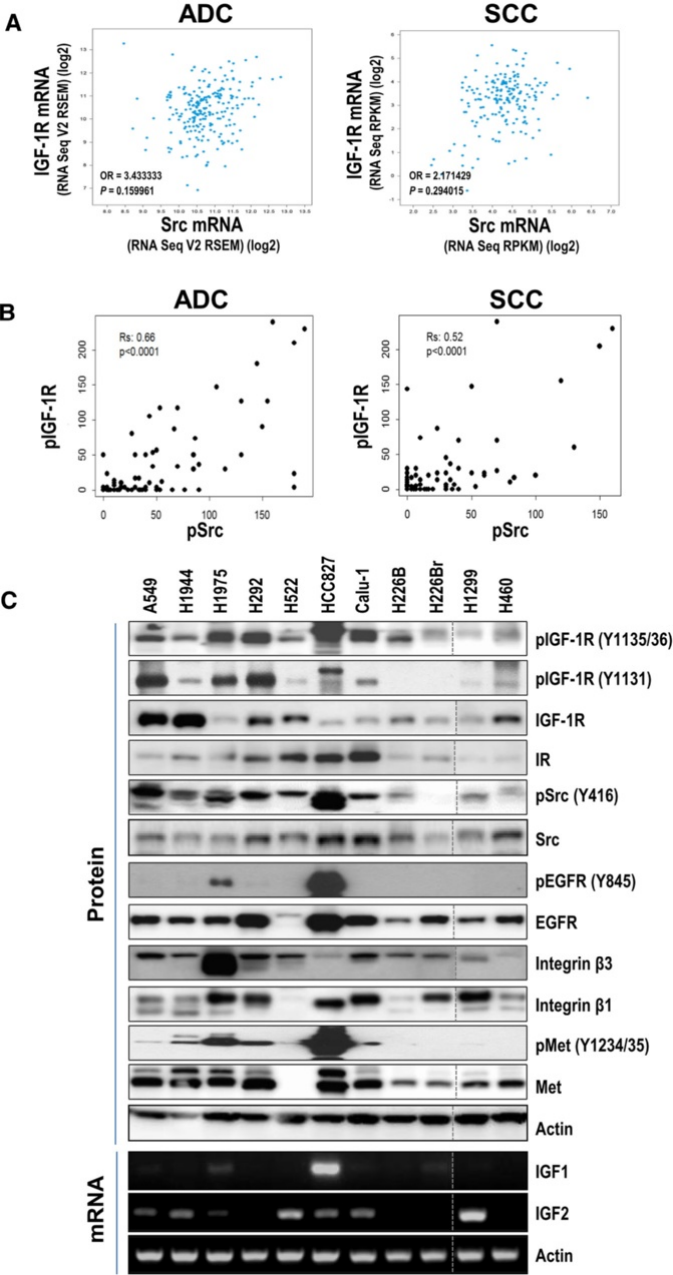
Co-activation of IGF-1R and Src in NSCLCs. (**a**) A comparison of mRNA levels of IGF-1R and Src in a dataset of lung cancer tissue specimens retrieved from cBioPortal (http://www.cbioportal.org/public-portal/). OR: odds ratio. (**b**) Correlation between pIGF-1R and pSrc in a tissue microarray. Rs: Spearman’s correlation coefficient. The significance of the correlation between pIGF-1R/IR and pSrc membrane levels was assessed using the Spearman Rank correlation test. (**c**) Protein and mRNA expression was examined *via* Western blot and RT-PCR analyses, respectively

We have previously demonstrated that pIGF-1R/IR levels are significantly higher in premalignant human lung epithelial tissue sample than in normal or reactive bronchial specimens and significantly correlated with the levels of IGF1 and IGF2 [18]. We have also shown that lung tumors can be formed by the lung-specific overexpression of IGF1 [18]. These results indicate the importance of tissue-derived IGF expression in the activation of the IGF-1R pathway at an early stage of lung carcinogenesis. We recently reported substantial levels of phosphorylated IGF-1R at tyrosine 1131 (tyrosine 1158 for IR) or tyrosines 1135 and 1136 (tyrosines 1162 and 1163 for IR) (pIGF-1R, hereafter) and IGF expression in tissue specimens from NSCLC patients [15, 16]. In the current study, we investigated whether the ligand-dependent activation of the IGF-1R occurs in NSCLC by analyzing previously studied tissue specimens from NSCLC patients. Unexpectedly, the pIGF-1R score was not correlated with the IGF1 (data not shown) or the IGF2 score (Additional file 1: Figure S1). Based on previous findings suggesting that Src can activate IGF-1R in a ligand-independent manner [19], we examined the relationship between IGF-1R and Src in NSCLC. A dataset retrieved from cBioPortal (http://www.cbioportal.org/public-portal/) revealed no significant correlation between the mRNA expression levels of IGF-1R and Src in adenocarcinoma (ADC; n = 532) and squamous cell carcinoma (SCC; n = 387) (Fig. 1a). In contrast, an immunohistochemistry (IHC) analysis of pIGF-1R and pSrc (Y416) (hereafter pSrc) expression in a human tissue microarray of NSCLC specimens (n = 353, consisting of 227 adenocarcinoma and 126 squamous cell carcinoma) [20] showed a significant correlation between pIGF-1R and pSrc levels regardless of histological subtype (Fig. 1b; Additional file 2: Figure S2). Consistent with our previous observations in NSCLC tissue samples, analysis of a panel of NSCLC cell lines with varying histologies and mutation status (Additional file 3: Table S1) further revealed that, except for the association between pIGF-1R and IGF1 in HCC827 cells, pIGF-1R levels were poorly correlated with ligand levels but were generally well correlated with pSrc expression in NSCLC cells (Fig. 1c; Additional file 4: Figure S3). pMet expression appeared to be correlated with pSrc expression, but the correlation between pIGF-1R and pSrc levels was stronger than the correlation between pMet and pSrc levels (Fig. 1c). We also observed high levels of membrane-associated receptors including EGFR, integrins β1 and β3, and/or c-Met, all of which are involved in Src activation [14], in most of the NSCLC cell lines. These findings indicate that Src and IGF-1R are co-activated in human NSCLC.

### Mutual phosphorylation of IGF-1R and Src in NSCLC cells

**Fig. 2:**
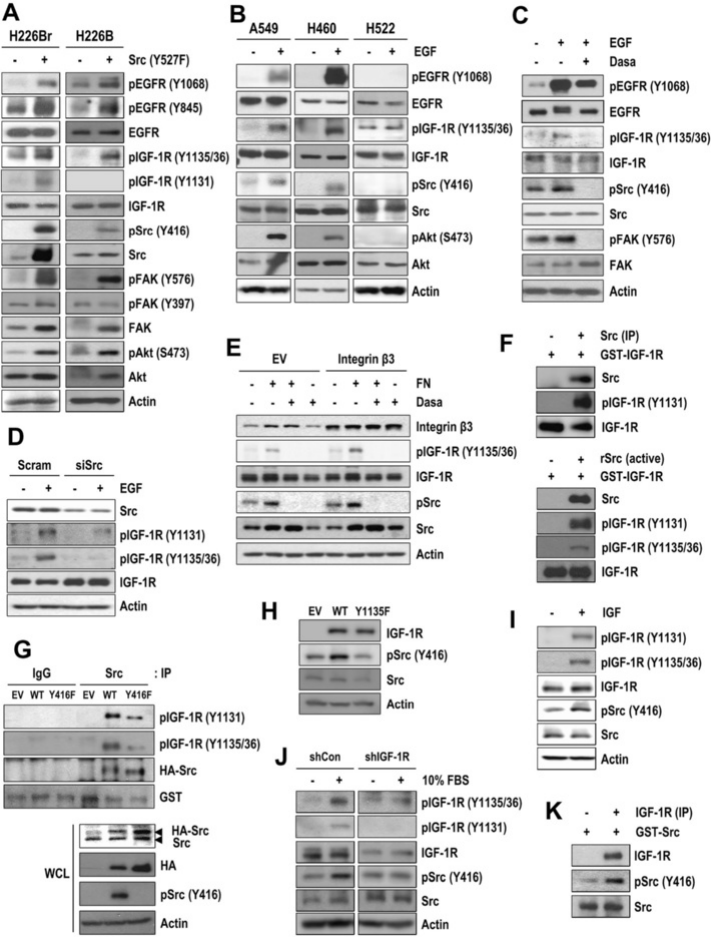
Transactivation of IGF-1R by activated Src. (**a**) H226B and H226Br cells were transiently transfected with empty or pcDNA3.1-Src (Y527F) vectors. (**b**) A549, H460, and H522 cells were serum-starved and then stimulated with EGF (50 ng/ml). (**c**) H520 cells were transfected with empty or pBabe-Puro EGFR WT vectors, treated with dasatinib (Dasa; 0.5 μM) for 2 h, and then stimulated with EGF (50 ng/ml) for 2 min. (**d**) A549 cells were transfected with scrambled (siCon) or Src siRNA (siSrc) and stimulated with EGF (50 ng/ml) for 5 min. (**e**) H226B cells were transfected with empty or pIRES2-EGFP-integrin β3 vectors, treated with dasatinib (Dasa; 0.5 μM) for 2 h, and then attached to fibronectin (FN)-coated dishes for 30 min. (**f**, **g**) *In vitro* Src kinase assay was performed using Src, either from recombinant protein (rSrc) or from immunoprecipitates (IP) from A549 cells untransfected (**f**) or from H226B cells transfected with wild-type or kinase-dead mutant Src (Y416F) (**g**), and recombinant IGF-1R (GST-IGF-1R) as a substrate. (**h**) H520 cells were transfected with empty, wild-type, or mutant IGF-1R (Y1135F)-expressing vectors. (**i**) A549 cells were serum-starved and then stimulated with IGF (100 ng/ml) for 5 minutes. (**j**) H1299 cells stably transfected with control- or IGF-1R shRNAs were stimulated with 10 % FBS for 5 minutes. (**k**) *In vitro* IGF-1R kinase assay was performed using IGF-1R immunoprecipitates (IP) from A549 cells and recombinant GST-Src as a substrate. The expression levels of the indicated proteins were determined by Western blot analysis

We assessed whether Src is involved in IGF-1R activation. Transfection with the constitutively active Src phosphorylated IGF-1R, EGFR (Y1068 and Y845), Src, and FAK (Y576, a Src-specific phosphorylation site [21]), and Akt (S473) but not FAK (Y397, an integrin signaling-induced autophosphorylation site [22]) or ERK1/2 in H226Br and H226B cells (Fig. 2a). We next assessed whether Src activation *via* various signaling pathways would affect IGF-1R phosphorylation. EGF stimulation increased EGFR, Akt, Src, and IGF-1R phosphorylation in A549 and H460 cells but not in H522, a low EGFR-expressing cell line [23] (Fig. 2b). This EGF-induced IGF-1R phosphorylation was suppressed by treatment with the clinically available small molecular Src inhibitor dasatinib [24] (Fig. 2c), by transfection with an siRNA against Src (Fig. 2d), and by treatment with the EGFR TKI erlotinib, but the IGF-1R TKI linsitinib exhibited relatively minimal effects on the suppression of EGF-induced IGF-1R phosphorylation (Additional file 5: Figure S4). Increased levels of pIGF-1R and pSrc were also observed when Src was activated through integrin signaling *via* attachment to fibronectin and/or the ectopic overexpression of integrin β3 (Fig. 2e; Additional file 6: Figures S5A and S5B). The integrin signaling-induced IGF-1R and Src phosphorylation was completely abolished by dasatinib treatment. These findings suggest that multiple membrane-associated receptors, including EGFR and integrin, can phosphorylate IGF-1R *via* Src activation.

Previous reports suggested that Src can directly phosphorylate IGF-1R at the sites of ligand-induced autophosphorylation [12, 13]. Consistent with this finding, *in vitro* kinase assays showed the ability of Src, derived from A549 cells or recombinant protein (rSrc), to phosphorylate recombinant IGF-1R protein (GST-IGF-1R) (Fig. 2f). Moreover, the Src immunoprecipitates from H226B cells transfected with wild-type Src showed greater IGF-1R phosphorylation than those from the kinase-dead Src (Y416F)-transfected cells (Fig. 2g). These findings indicated that Src can directly phosphorylate IGF-1R, but indirect mechanisms (as a consequence of an autocrine mechanism or the activation of another kinase) may be also involved in Src-induced IGF-1R phosphorylation.

We next assessed the potential involvement of IGF-1R in Src phosphorylation. To this end, we constructed a mutant IGF-1R that replaced tyrosine 1135 with phenylalanine (Y1135F). In contrast to the wild-type receptor, this mutant was unresponsive to IGF-stimulated IGF-1R tyrosine phosphorylation [25], confirming the importance of the site for receptor activity. Transfection with wild-type IGF-1R but not a mutant IGF-1R (Y1135F) (Fig. 2h) or stimulation with IGF-1 (Fig. 2i) or 10 % FBS (Fig. 2j, left) induced Src phosphorylation (Additional file 6: Figure S5C–S5E). The FBS-induced Src phosphorylation was effectively attenuated by transfection with a shRNA against IGF-1R (Fig. 2j, right; Additional file 6: Figure S5E). An *in vitro* kinase assay showed that IGF-1R immunoprecipitated from A549 cells phosphorylated Src (Fig. 2k; Additional file 6: Figure S5F). These findings revealed the ability of IGF-1R to phosphorylate Src. Collectively, these results indicated the mutual phosphorylation of IGF-1R and Src in NSCLC cells.

### Src-dependent activation of IGF-1R downstream signaling effectors in high-pSrc-expressing NSCLC cells after treatment with IGF-1R TKIs

**Fig. 3:**
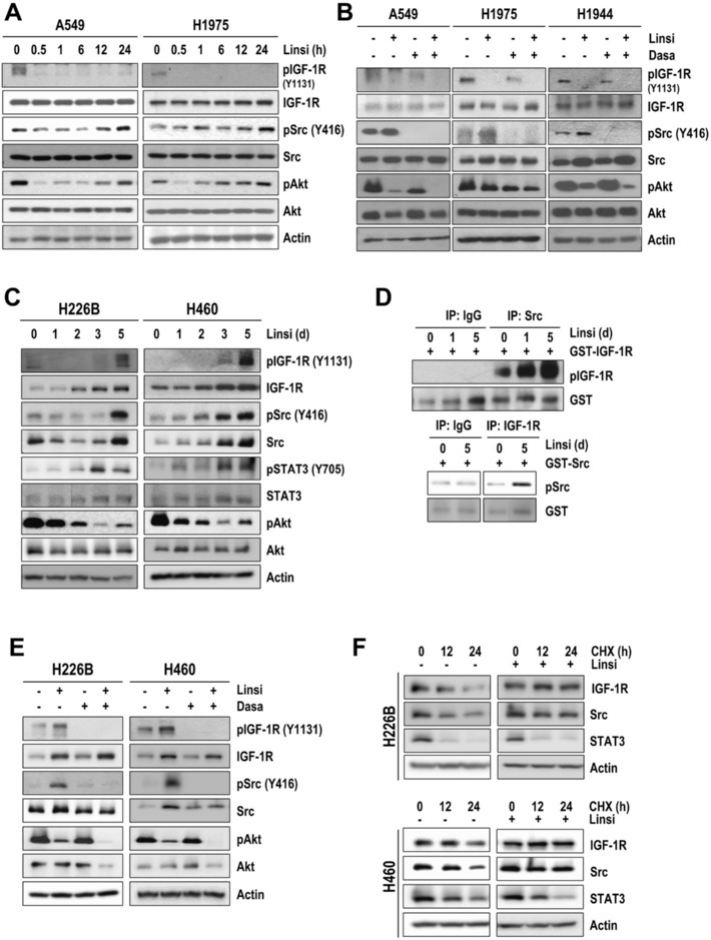
Linsitinib-mediated reciprocal co-activation of IGF-1R and Src confers linsitinib resistance. (**a**, **b**, **c**, **e**, and **f**) A549 and H1975 cells were treated with linsitinib (Linsi) (2 μM) alone (**a**) or in combination with dasatinib (Dasa) (100 nM) (**b**) for the indicated hours (**a**) or for 1 day (**b**) in the presence of 10 % FBS. H226B and H460 cells were subject to daily treatment with linsitinib (1 μM) for 1-5 days (**c**, **f**) or 5 days (**e**) either alone or in combination with dasatinib (100 nM) for the last 1 day (**e**). Cells treated with linsitinib for 5 days were subjected to cycloheximide treatment for the indicated time points (**f**). The indicated protein expression was determined by Western blot analysis. *P < 0.05. (**d**) *in vitro* kinase assay was performed using Src and IGF-1R immunoprecipitates and GST-IGF-1R or GST-Src recombinant proteins as substrates

We then assessed the effect of Src activity on the efficacy of IGF-1R TKIs in a subset of high-pSrc-expressing (A549, H1944, H1975, H292, HCC827) and low-pSrc-expressing (H226B, H226Br, H1299, H460 and Calu-1) NSCLC cell lines based on densitometric quantification of phosphorylated Src blots (Additional file 7: Figure S6). Treatment with linsitinib effectively suppressed IGF-1R phosphorylation at both Y1135/36 and Y1131 (Additional file 8: Figure S7). As monitored the kinetics of IGF-1R, Akt and Src phosphorylation, in spite of sustained dephosphorylation of IGF-1R by linsitinib treatment, Akt, EGFR, and Src, but not ERK, were rapidly dephosphorylated but gradually rephosphorylated in a time-dependent manner (Fig. 3a; Additional files 9 and 10: Figure S8A and S9). Treatment with linsitinib also increased in the Src-specific phosphorylation of EGFR at tyrosine 845, confirming induction of Src activation by linsitinib treatment (Additional file 10: Figure S9). We further discovered that a combined treatment with linsitinib and dasatinib suppressed pIGF-1R, pSrc, and pAkt levels (Fig. 3b). These findings suggest that high-pSrc-expressing NSCLC cells can bypass IGF-1R and activate downstream molecules *via* Src activity.

### Linsitinib treatment-induced stabilization of IGF-1R and Src protein leads to the reciprocal co-activation of IGF-1R and Src in low-pSrc-expressing NSCLC cells

Based on the mutual activation of IGF-1R and Src and the Src-dependent activation of Akt in high pSrc-expressing cells after linsitinib treatment, we speculated that cells with a relatively low level of active Src may display high levels of linsitinib sensitivity. However, when we compared the linsitinib sensitivity of a subset of NSCLC cell lines with high or low levels of pSrc expression, we observed a statistically significant inverse correlation between pSrc levels and linsitinib sensitivity (Additional file 11: Figure S10). We then monitored the effects of linsitinib on IGF-1R and Src phosphorylation in low-pSrc-expressing H226B and H460 cells during 5 days of daily treatment with linsitinib (1 μM). We found transient decreases in pIGF-1R and pSrc levels followed by time-dependent increases in pIGF-1R, pSrc, pSTAT3, and pAkt in the linsitinib-treated H226B cells (Fig. 3c; Additional file 9: Figure S8B). *In vitro* kinase assays showed that IGF-1R and Src in the linsitinib-treated cells had increased capacity for mutual phosphorylation (Fig. 3d). Moreover, combined treatment with linsitinib and dasatinib effectively suppressed the linsitinib-induced activation of IGF-1R, Src, and Akt (Fig. 3e). The linsitinib treatment of H460 and Calu-1 cells also showed an initial decrease followed by a time-dependent increases in IGF-1R, Src, and Akt phosphorylation (Fig. 3c; Additional file 12: Figure S11), which was blocked by the dasatinib treatment (Fig. 3e; Additional file 12: Figure S11). Combined treatment with another SFK inhibitor PP2 [26] also decreased linsitinib-induced IGF-1R, Src, and Akt phoshsphorylation in H460 cells (Additional file 13: Figure S12). These findings suggest that linsitinib treatment augmented the reciprocal co-activation of IGF-1R and Src in low-pSrc-expressing NSCLC cells. Notably, IGF-1R and Src protein expressions were markedly increased in H226B and H460 cells after 3 days of treatment with linsitinib (Fig. 3c). Therefore, we assessed whether the increased levels of IGF-1R and Src proteins in linsitinib-treated cells were due to increased protein synthesis or stability by measuring these protein levels after the linsitinib-treated cells were treated with cycloheximide (CHX). The linsitinib-treated H226B and H460 cells showed a markedly decreased turnover of IGF-1R and Src proteins after 24 h of cycloheximide treatment compared with those treated with vehicle (Fig. 3f). These findings suggested that linsitinib treatment stabilized IGF-1R and Src proteins in low-pSrc-expressing NSCLC cells, ultimately leading to the enhanced reciprocal activation of IGF-1R and Src. Interestingly, in spite of blockade of EGFR phosphorylation, the increases in total and phosphorylated Src and total EGFR expression were found in A549 cells treated with an EGFR TKI erlotinib for 3 days, suggesting that these phenomenon might not be specific to the IGF-1R targeted agents and NSCLC cells may also be able to bypass EGFR by activating Src (Additional file 14: Figure S13).

### Src activity mediates *de novo* linsitinib resistance in NSCLC cells

**Fig. 4:**
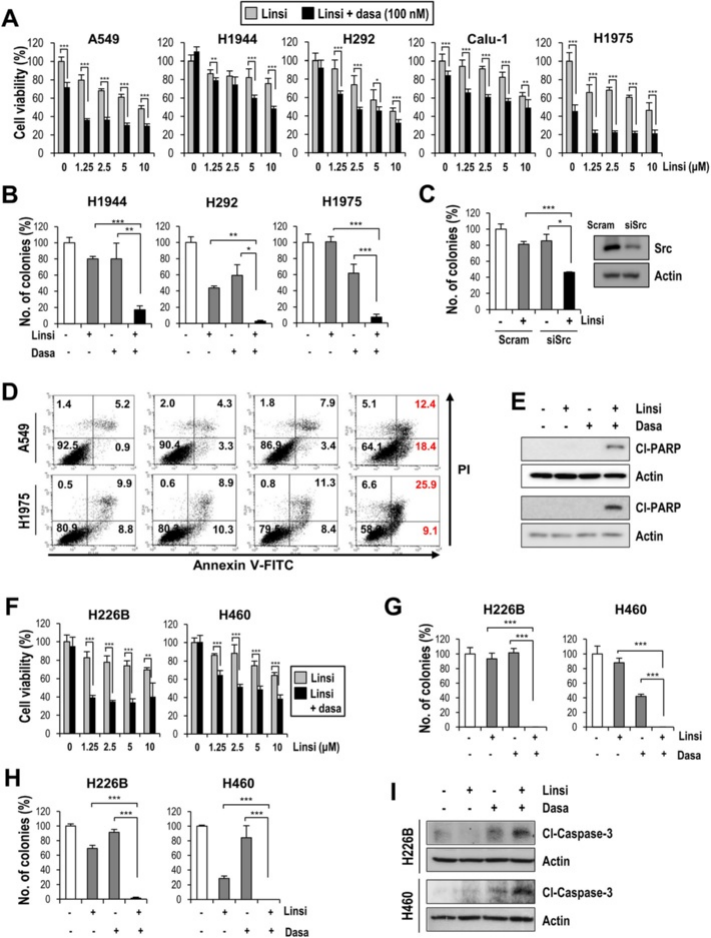
Targeting Src overcomes IGF-1R TKI resistance *in vitro* both in high-pSrc-expressing and low-pSrc-expressing NSCLC cells. The effects of linsitinib, either alone or in combination with dasatinib, on viability (**a** and **f**), anchorage-dependent (**b** and **g**), and anchorage-independent (**h**) colony formation, and apoptosis (**d**, **e** and **i**) were determined. The induction of apoptosis was analyzed by flow cytometry after staining with annexin V-FITC and PI (**d**) and Western blot analysis on PARP and caspase-3 cleavages (**e** and **i**). The bars represent the means ± SD; *P < 0.05, **P < 0.01, and ***P < 0.001. (**c**) Cells were transfected with scrambled or Src siRNAs. Transfected cells were seeded into 6-well plates at a density of 150 cells/well to evaluate the inhibitory effect of linsitinib on the anchorage-dependent growth as described in Supplemental Information. The bars represent the means ± SD; *P < 0.05 and ***P < 0.001

We then assessed the sensitivity of NSCLC cells to linsitinib in medium containing low (1 %) or high (10 %) fetal bovine serum (FBS), which contains various ligands for the membrane-associated receptors [27]. Linsitinib treatment decreased viability (Additional file 15: Figure S14A) and colony forming ability (Additional file 15: Figure S14B) and induced apoptosis (Additional file 15: Figure S14C) in both high-pSrc-expressing and low-pSrc-expressing NSCLC cells more effectively in low serum conditions than in high serum conditions. The inhibitory effects of linsitinib on the viability (Fig. 4a) and colony-forming ability (Fig. 4b) of NSCLC cells in high serum conditions were significantly enhanced by dasatinib treatment. The enhanced inhibitory effects of linsitinib on the colony formation ability were also observed in A549 cells after Src expression was knocked-down by siRNA transfection (Fig. 4c). Furthermore, combined treatment with linsitinib and dasatinib induced apoptosis in A549 and H1975 cells in a substantially more potent manner than either single drug treatment, as shown by flow cytometry and Western blot analysis of PARP cleavage (Fig. 4d and e; Additional file 16: Figure S15).

In accordance with our above findings from the high pSrc-expressing NSCLC cells, linsitinib treatment decreased the viability of low pSrc-expressing NSCLC cells more effectively under low-serum conditions than high-serum conditions (Additional file 15: Figure S14A and S14B). Similar results were observed after treatment with another IGF-1R TKI, AG1024 (Additional file 17: Figure S16). In addition, combined treatment with linsitinib and dasatinib suppressed cell proliferation (Fig. 4f) and anchorage-dependent (Fig. 4g) and anchorage-independent colony formation (Fig. 4h) and increased caspase-3 cleavage (Fig. 4i) in low pSrc-expressing H226B and H460 cells more potently than either drug alone.

Enhanced effects of co-targeting IGF-1R and Src on viability (Additional file 18: Figure S17A), anchorage-dependent (Additional file 18: Figure S17B) and anchorage-independent colony formation (Additional file 18: Figure S17C) were also observed in both high-pSrc-expressing and low-pSrc-expressing NSCLC cells when another pharmacologic SFK inhibitor, PP2 [26], was combined with linsitinib. Notably, co-targeting Src and IGF-1R effectively suppressed the colony formation of EGFR TKI-resistant NSCLC cells (H1975, PC-9/GR and PC-9/ER) [28] (Additional file 18: Figure S17D). These results suggest that Src antagonism may broadly increase linsitinib sensitivity in NSCLC cells regardless of IGF-1R and Src activity as well as EGFR TKI resistance.

### Targeting Src overcomes IGF-1R TKI resistance *in vivo*

**Fig. 5:**
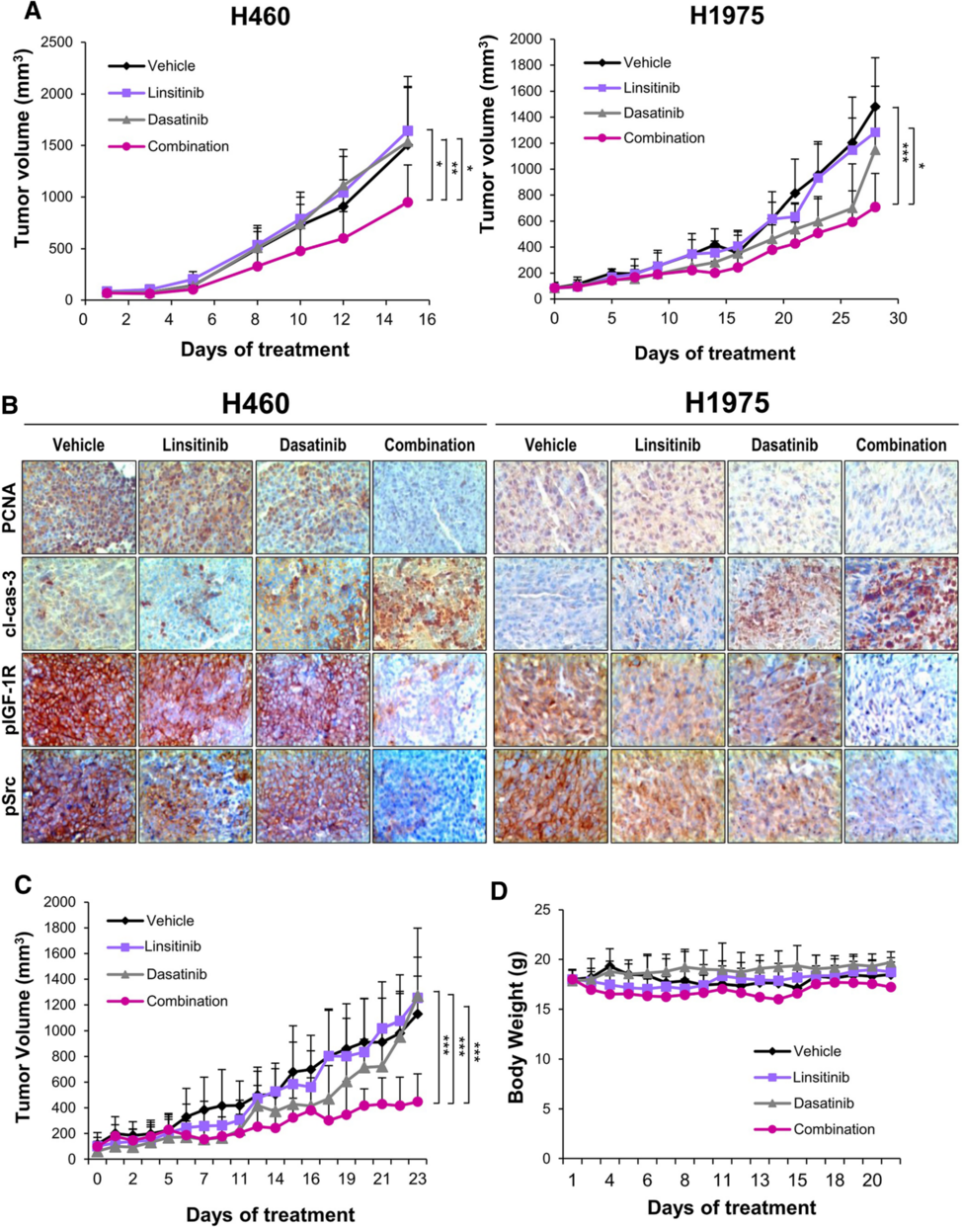
Effects of co-targeting IGF-1R and Src on the growth of xenograft tumors and patient-derived heterotransplants. H460 and H1975 cells (**a**) or tumors derived from a NSCLC patient (**c**) were subcutaneously injected into athymic nude mice or NOD/SCID mice. Mice bearing tumors were treated with linsitinib (Linsi), dasatinib (Dasa), or their combination. (**a** and **c**) The data are presented as the mean tumor volume ± SD for the indicated times. *P < 0.05, **P < 0.01, and ***P < 0.001. (**b**) The tumors were subjected to IHC analysis to determine the levels of PCNA, pIGF-1R, pSrc, and cleaved caspase-3 (cl-Caspase3) staining. Representative tumors from each group are shown. (**d**) Body weight of the treated mice was monitored every 2 days

We analyzed the effects of the combined inhibition of IGF-1R and Src on tumor growth *in vivo*. As predicted in the *in vitro* studies, we observed a potent antitumor effect of combination treatment in xenograft tumors of both high-pSrc-expressing (H1975) and low- pSrc-expressing (H460) (Fig. 5a) NSCLC cells. At the end of the study, there was a statistically significant difference in the tumor volumes in the vehicle-, linsitinib-, and dasatinib-treated groups and the group treated with the combination of linsitinib and dasatinib. An IHC analysis of the xenograft tumors tissues showed that the combined treatment with linsitinib and dasatinib resulted in obviously increased levels of active caspase-3 and decreased levels of PCNA, pIGF-1R, and pSrc compared with those observed after vehicle or single agent treatments (Fig. 5b). Finally, we assessed the benefit of combined IGF-1R and Src inhibition in NSCLC tumors obtained from a human patient. Consistent with the results from xenograft tumors of NSCLC cell lines, a potent combinatory antitumor effect became apparent over the course of the treatment and was statistically significant after 23 days of combined treatment (Fig. 5c). Mice receiving the combined treatment showed a slight but not statistically significant decrease in body weight compared with the control or single-treatment groups (Fig. 5d). Collectively, these findings suggest that co-targeting the IGF-1R and Src is an effective therapeutic strategy for the treatment of NSCLC.

## Discussion

**Fig. 6:**
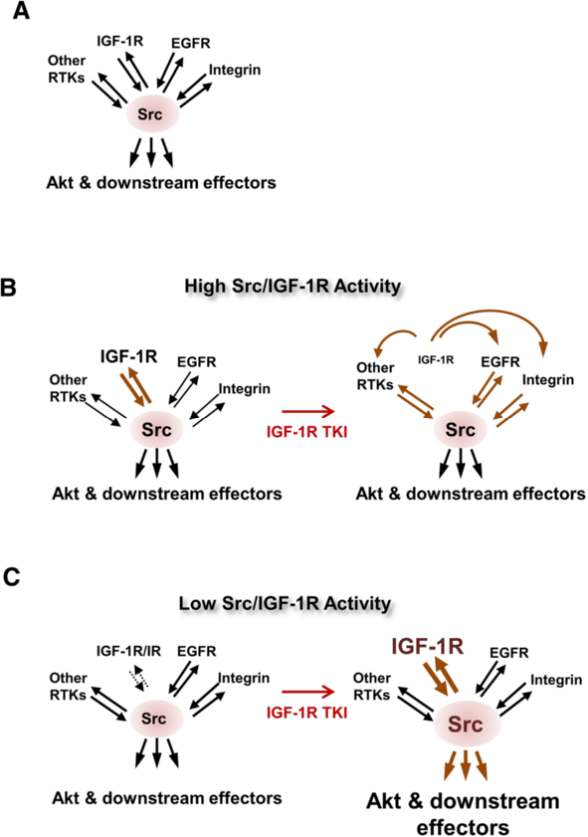
Schematic model of the IGF-IR TKI resistance. (**a**) Src, a common downstream node of various membrane-associated receptors, stimulates downstream signaling molecules. (**b**) IGF-1R and Src are simultaneously co-activated through mutual phosphorylation in human NSCLCs. The suppression of IGF-1R with a TKI reprograms high- pSrc-expressing NSCLC cells to stimulate downstream signaling effectors *via* alternative pathways of Src activation through various plasma membrane-associated receptors resulting in drug resistance. (**c**) The IGF-1R TKI-mediated suppression of IGF-1R in low-pSrc-expressing NSCLC cells induces the posttranslational reprogramming of the cellular machinery to decrease the turnover of the IGF-1R and Src proteins, which in turn augments reciprocal co-activation of Src and IGF-1R, resulting in drug resistance

Recent studies have demonstrated the signaling plasticity of tumor cells in which the blockade of specific targets by anticancer drugs stimulates multiple compensatory signaling molecules and provides an adaptive survival potential to tumor cells, ultimately conferring drug resistance. In this work, we show that 1) Src acts as a common downstream node of multiple membrane-associated receptors, including IGF-1R, EGFR, and integrin, stimulating downstream molecules, such as Akt, to sustain cell viability (Fig. 6a); 2) Src and IGF-1R are reciprocally co-activated at high levels in the majority of NSCLC cells. The suppression of IGF-1R with a TKI leads to the utilization of alternative pathways of Src activation, most likely *via* various membrane-associated receptors, resulting in resistance to the drug (Fig. 6b); 3) suppression of IGF-1R with a TKI induces the posttranslational reprogramming of cells to activate cellular machinery to stabilize the Src and IGF-1R proteins in NSCLC cells with low levels of IGF-1R and Src co-activation, leading to the enhanced reciprocal co-activation of Src and IGF-1R and resistance to the drug (Fig. 6c). We also show that targeting Src universally sensitizes NSCLC cells to IGF-1R TKIs. These results, together with previous reports and our current findings showing the simultaneous over-expression/activation of multiple membrane-associated receptors in NSCLCs [29–31], suggest that combination regimens of molecularly targeted agents against IGF-1R and Src have a great potential for anticancer therapies.

The IGF-1R pathway plays a major role in mediating the activation of prosurvival pathways and contributes to resistance to various anticancer therapies, including chemotherapies, radiation therapy, and molecular targeted therapies, in multiple cancer types [5–7, 11, 32]. These findings indicate that IGF-1R-targeted therapies represent a promising single-agent or combination regimen. However, NSCLC cells appear to exhibit resistance to the IGF-1R-targeted agents through various mechanisms. With respect to IGF-1R mAb resistance, our laboratory has shown that the inactivation of IGF-1R by the IGF-1R mAb resulted in enhanced signaling through IGF-dependent integrin/Src signaling and the Akt/mTOR-mediated protein synthesis of EGFR and Akt, leading to drug resistance [33, 34]. IR has also been suggested to mediate intrinsic and/or acquired resistance to IGF-1R mAbs [30, 35]. In this regard, dual IGF-1R/IR TKI regimens may be more effective in blocking the IGF-1R pathway in patients with NSCLC. However, the dual IGF-1R/IR TKI linsitinib has shown moderate efficacy and/or severe toxicity in multiple preclinical and clinical studies for various types of cancer^3^ [36, 37]. Furthermore, the major components of the IGF-1R pathway, such as pIGF-1R, pAkt, pERK, pIRS, and pS6K, could not predict response to linsitinib [38]. Therefore, identifying and targeting the mechanism of resistance against the IGF-1R TKIs would be effective and clinically practical in controlling prosurvival pathways.

*De novo* and acquired resistance to inhibitors of RTK have been suggested to occur due to the mutation, amplification, and increased/decreased expression of signaling molecules involved in multiple counter-regulatory pathways [39]. However, unlike EGFR, neither activating mutations nor gene amplifications of IGF-1R are common [40]. We have demonstrated that the activation of the IGF-1R pathway through the increased expression of tissue-derived IGFs and the loss of IGFBP-3 is an early event in lung carcinogenesis [18, 41]. However, an analysis of previously studied tissue specimens from NSCLC patients and a panel of NSCLC cell lines revealed no correlation between the expression levels of pIGF-1R and IGFs. The previous reports, including; 1) the mutation and/or overexpression of various molecules involved in Src activation, such as EGFR, KRas, and HER2 [16, 29], has been implicated in the mechanism of resistance to IGF-1R TKIs [16]; and 2) the aberrant overexpression and activation of IGF-1R and Src in many NSCLCs [42], and our current results, indicating; 1) a strong positive correlation between pSrc and pIGF-1R in a large TMA of NSCLC tissues and in the majority of NSCLC cell lines; 2) the suppression of EGFR- or integrinβ3 -mediated activation of IGF-1R *via* dasatinib treatment; and 3) mutual direct phosphorylation between Src and IGF-1R, suggested that Src acts as a key node that link signaling from the cross-talk of several membrane-associated receptors to the ligand-independent activation of IGF-1R in NSCLC.

We then hypothesized that Src plays a critical role in resistance to IGF-1R TKIs in NSCLC cells. In support of the notion, combined treatment with linsitinib and dasatinib was more efficacious in suppressing the proliferation and colony-forming ability of high-pSrc-expressing NSCLC cells than single drug treatments. We further expected that low-pSrc-expressing cells would be more sensitive to IGF-1R TKIs. However, low-pSrc-expressing NSCLC cells showed significantly weaker sensitivity to IGF-1R TKIs than high-pSrc-expressing NSCLC cells. Our subsequent studies provide evidence that the treatment of low-pSrc-expressing cells with an IGF-1R TKI reprograms the cells to stabilize IGF-1R and Src proteins, leading to the enhanced reciprocal coactivation of IGF-1R and Src. Moreover, combined treatment with linsitinib and dasatinib resulted in significantly enhanced antiproliferative effects in these NSCLC cell lines. Importantly, co-targeting IGF-1R and Src exhibited apoptotic activities *in vitro* in both high-pSrc-expressing and low-pSrc-expressing NSCLC cell lines as well as *in vivo* in immunodeficient mice bearing xenograft tumors of these cell lines and in those bearing heterotransplant xenogrft tumors from a patient with NSCLC. Collectively, these findings suggest a potential benefit of targeting Src in preventing NSCLC cell resistance to IGF-1R TKIs regardless of IGF-1R or Src activity.

Notably, the combination of linsitinib and dasatinib induces apoptosis in H1975, PC-9R, and PC-9/ER cells with acquired resistance to EGFR TKIs *in vitro* and causes substantial tumor shrinkage in mice harboring H1975 xenograft tumors *in vivo. EGFR*-mutant tumors are known to have an increased sensitivity to the first-generation EGFR TKIs gefitinib and erlotinib. However, a secondary T790M mutation promotes the acquisition of resistance to EGFR TKIs in *EGFR-*mutant tumors. Recent studies suggest that second- and third-generation EGFR inhibitors are more potent than gefitinib or erlotinib (at least in pre-clinical models) against the drug-resistant mutation [43]. In addition, several combination strategies, including the combination of EGFR TKIs with inhibitors of downstream EGFR targets, such as PI3K or STAT5, have been tested in preclinical models of *EGFR-*mutant NSCLCs [44]. Although these strategies are promising, their clinical efficacy remains to be evaluated. Hence, there is a need for alternative strategies to address the problem of resistance to EGFR TKIs. In this regard, our current findings suggest that the co-inhibition of IGF-1R and Src may provide a substantial therapeutic advantage for patients with NSCLC with acquired resistance to EGFR TKIs as well.

## Conclusions

We have demonstrated a potential biological interaction between IGF-1R and Src signalings in NSCLC. We also show that this interaction interferes with the therapeutic activities of IGF-1R TKIs and that the addition of a Src inhibitor is effective in overcoming resistance to IGF-1R TKIs. Because the clinical development of TKIs of IGF-1R and Src is ongoing [45, 46], our findings directly impact the current clinical management of patients with NSCLC. It is noteworthy that a phase I trial of solid tumors treated with XL228 (a small molecule inhibitor targeting Src family kinases, IGF-1R, Aurora A, and Bcr-Abl in the low nanomolar range [47]) has shown promising preliminary clinical results in melanoma and solid tumors including NSLCLC [48]. Considering the extensive toxicities of Src inhibitors and IGF-1R TKIs [49, 50], further studies are warranted to evaluate the efficacy of co-targeting IGF-1R and Src in additional preclinical and clinical settings.

## Methods

### Cell culture

Human NSCLC cells (A549, H1975, H292, H522, HCC827, Calu-1, H520, H1299, H460, H1993, H2122, and H1703) were purchased from ATCC (Manassas, VA, USA). Other NSCLC cells were kindly provided by Dr. John V. Heymach (MD Anderson Cancer Center, Houston, TX, USA). PC-9R cells (gefitinib-resistant PC-9 cells) were kindly provided by Dr. Mien-Chie Hung (MD Anderson Cancer Center, Houston, TX, USA). PC-9/ER (H) cells (erlotinib-resistant PC-9 cells) were kindly provided by Dr. Jae Cheol Lee (Asan Medical Center, Seoul, Republic of Korea).The cells were maintained in RPMI 1640 medium supplemented with 10 % fetal bovine serum (FBS) and antibiotics. The cells were incubated at 37 °C with 5 % CO_2_ in a humidified atmosphere.

### Reagents

Linsitinib (OSI-906) was provided by OSI Pharmaceuticals, LLC (Melville, New York, USA) or purchased from Selleckchem (Houston, TX, USA). PP2 was purchased from EMD Chemicals (Gibbstown, NJ, USA). Dasatinib was purchased from LC laboratories (Woburn, MA, USA) or the pharmacy in the MD Anderson Cancer Center. Antibodies against pIGF-1R/IR (Y1131), pIGF-1R/IR (Y1135/36), IGF-1R, IR, pEGFR (Y1068), pEGFR (Y845), Src, pSrc (Y416), Met, pAkt, Akt, pERK1/2 (p42/44), ERK and cleaved caspase-3 were purchased from Cell Signaling Technology (Danvers, MA, USA). Antibodies against cleaved PARP, pFAK (Y397), FAK, integrin β1, and integrin β3 were purchased from BD Biosciences (San Jose, CA, USA). Antibodies against actin, IGF-1R (C-20), IR, and EGFR (1005) were purchased from Santa Cruz Biotechnology (Santa Cruz, CA, USA). Antibodies against pIR/IGFR (Y1162/63) and pFAK (Y576) were purchased from Invitrogen (Carlsbad, CA, USA). Chemicals unless otherwise indicated were purchased from Sigma-Aldrich (St. Louis, MO, USA). The pIRES2-EGFP-integrin β3 expression vector was kindly provided by Dr. Jung Weon Lee (Seoul National University, Seoul, Republic of Korea). The pBabe-Puro EGFR WT plasmid was purchased from Addgene (catalog # 11011, Cambridge, MA, USA) [51]. The mutant Src (Y527F) expression vector was kindly provided by Dr. Faye M. Johnson (MD Anderson Cancer Center, Houston, TX, USA).

### MTT assay

Cells were treated with increasing concentrations of linsitinib or SFK inhibitors (dasatinib or PP2), alone or in combination, for 3 days. Drugs were diluted in 1 % or 10 % FBS-containing media. Cell viability was analyzed by the MTT assay. Data were presented as a percentage of the vehicle-treated control group.

### Anchorage-dependent growth inhibition

Cells were seeded into 12- or 6-well plates at a density of 80–150 cells/well and were incubated for 24 h. Cells were treated with linsitinib (2 μM) and dasatinib (0.1 μM) or PP2 (2 μM), either alone or in combination, for 12 ~ 14 days. After incubation, cells were fixed with 100 % methanol for 10 mins at room temperature. Cells were further stained with 0.002 % crystal violet solution or hematoxylin for 1 h at room temperature, and then washed with PBS 3 ~ 5 times. Stained colonies were photographed and counted.

### Anchorage-independent growth inhibition

Before the experiment, 1 % base agar was made by diluting 4 % sterile low-melting agar solution with 10 % RPMI and pouring 1 ml of the mixture in 12-well plates. H460, H226B, and H226Br cells were harvested and diluted in the medium at a density of 7 × 10^3^ cells/ml (final concentration of 2 × 10^3^ cells/well). Cells were mixed with sterile 1 % agar solution (final concentration of 0.4 %) and immediately poured onto the base agar. After the agar was completely solidified, test samples diluted in 0.5 ml of complete medium were added to the cells. Cells were further incubated for 12 days at 37 °C and 5 % CO_2_. The colonies were stained with MTT solution (final 200–500 μg/ml). Stained colonies were photographed and counted.

### Cell cycle analysis

Cells (3 × 10^5^ cells/dish in 60 mm dishes) were incubated with test samples for 48 h. All adherent or floating cells were collected and washed twice with PBS. Cells were fixed with 100 % methanol overnight. Fixed cells were washed with PBS, and then stained with 50 μg/ml propidium iodide (PI) solution containing 50 μg/ml RNase A for 30 min at room temperature. Fluorescence intensity was analyzed using a FACSCalibur® flow cytometer (BD Biosciences, San Jose, CA, USA). The percentages of the distributions in distinct phases of the cell cycle were determined using the ModFIT LT V2.0 software.

### Annexin V/PI double staining

A549, H1975, PC-9R, and PC-9/ER (H) cells were treated with linsitinib and dasatinib alone or in combination for 3 days. Adherent and floating cells were collected and washed with PBS. Cells were diluted with 1X binding buffer at a density of 1 × 10^5^ cells/0.1 ml and then stained with Annexin V or PI using an Annexin V/PI double staining kit (BD Biosciences) according to the manufacturer’s instruction. Stained cells were analyzed by flow cytometry.

### Western blot analysis

Cell lysates were prepared with modified RIPA lysis buffer (50 mM Tris-HCl (pH 7.4), 150 mM NaCl, 1 mM EDTA, 0.25 % Sodium deoxycholate, 1 % Triton X-100, protease inhibitor cocktail (Roche Applied Science, Indianapolis, IN, USA), and phosphatase inhibitor cocktail (Roche)). Crude lysates were centrifuged at 13,000 rpm for 30 min at 4 °C. Supernatants were collected, and protein concentration was determined by BCA assay (Thermo Fisher Scientific, Waltham, MA, USA). Equal amounts (10–20 μg) of lysates were subjected to 8–10 % SDS-PAGE. Separated proteins were transferred onto a PVDF membrane (Bio-Rad Laboratories, Hercules, CA, USA). Membranes were blocked with blocking buffer (5 % BSA in PBS containing 0.1 % Tween-20 (PBST)) for 1 h at room temperature and followed by incubation with primary antibodies diluted in 5 % BSA in PBST (1:1000) overnight at 4 °C. Membranes were washed three times with PBST, and incubated with the corresponding secondary antibodies diluted in 3 % non-fat dry milk in PBST (1:5000) for 1–2 h at room temperature. Membranes were washed three times with PBST and were visualized using an enhanced chemiluminescence (ECL) detection kit (Thermo Fisher Scientific).

### RT-PCR

Total RNA isolation and RT-PCR were performed as described previously [5] using the following primer sequences: (sense) 5’-TCTTGAAGGTGAAGATGCACACCA-3’ and (antisense) 5’-AGCGAGCTGACTTGGCAGGCTTGA-3’ for IGF1; (sense) 5’-CATCGTTGAGGAGTGCTGTTT-3’ and (antisense) 5’-GTCTTGGGTGGGTAGAGCAAT-3’ for IGF2; and (sense) 5’-ACTACCTCATGAAGATC-3’ and (antisense) 5’-GATCCACATCTG CTGGAA-3’ for actin. PCR was performed under the following conditions: 95 °C for 5 min (initial denaturation), followed by 28 or 35 cycles of 95°C for 30 sec, 55 (for IGF2 and Actin) or 58 °C (for IGF1) for 30 sec, and 72 °C for 30 sec, and a final elongation cycle of 72 °C for 7 min. PCR products were resolved on a 2 % agarose gel containing RedSafe nucleic acid staining solution (Intron Biotechnology, Seongnam-si, Kyunggi-do, Republic of Korea) and visualized using a Gel Doc EZ System (Bio-Rad Laboratories).

### Transfection

Transfection was conducted with lipofectamine 2000 (Invitrogen) according to the manufacture’s recommended procedure.

### *In vitro* kinase assay

A549 cells were lysed in modified RIPA buffer, and immunoprecipitation was performed via incubation with 0.8 μg of anti-c-Src antibody and 20 μl of protein G agarose. After overnight incubation at 4 °C, the immunoprecipitates were washed three times with lysis buffer, washed three times with kinase buffer (20 mM HEPES (pH 7.5) 10 mM MgCl_2,_ 0.5 mM EGTA, 2 mM DTT, 0.5 mM Na_3_VO_4_, and 0.5 M NaF), suspended in 30 μl kinase buffer containing ATP (20 μM) and 200 ng of purified GST-IGF-1R (aa959–1367) as a substrate, and incubated for 30 min at 37 °C for *in vitro* kinase assays. The reaction products were analyzed via Western blotting with a phospho-IGF-1R (Y1131) antibody.

For IGF-1R kinase assays, 200 ng of purified GST-c-Src (aa1-536) was reacted with immunoprecipitates against IGF-1Rβ in a kinase buffer containing ATP (20 μM) at 37 °C for 30 min and then analyzed via Western blotting with a phospho-Src family (Tyr 416) antibody. Src and IGF-1R kinase activity in linsitinib-treated H460 cells were also determined as described above.

### Protein degradation assay

To investigate the effects of linsitinib on IGF-1R and Src protein stability, cells maintained in linsitinib for 5 days were treated with cycloheximide (CHX, 50 μg/ml) for the indicated time, and the total IGF-1R and Src levels were analyzed via Western blotting.

### *In vivo* tumor xenograft model

All animal procedures were performed in accordance with a protocol approved by the Seoul National University Institutional Animal Care and Use Committee (approval Nos. SNU-130426-9 and SNU-130820-6). Mice were fed standard mouse chow and water *ad libitum* and housed in temperature- and humidity-controlled facilities with a 12-hour light/12-hour dark cycle. H1975 and H460 cells (1 × 10^7^ cells) were subcutaneously injected into the left and right flank of each six week-old female athymic mouse on day 0. For the patient-derived tumor xenograft (PDX) experiment, tumors that were passed 3 times in mice were minced into 2-mm^3^ pieces and subcutaneously inoculated into NOD/SCID mice. When the tumor volume reached approximately 100–150 mm^3^, the mice were randomly divided into four groups and treated with linsitinib (25 or 50 mg/kg, oral gavage), dasatinib (10 or 25 mg/kg, oral gavage), or a combination of linsitinib and dasatinib six times per week for 3 weeks. Linsitinib was dissolved in a 25 mM tartaric acid solution. Dasatinib powder was dissolved in an 80 mM citric acid solution or a 100 mg dasatinib tablet was suspended in PBS. Tumor growth was determined by measuring the short and long diameters of the tumors with a caliper every 2 days, and body weight was measured to monitor drug toxicity. The tumor volume was calculated using the following formula: tumor volume (mm^3^) = (short diameter)^2^ × (long diameter) × 0.5.

### Immunohistochemistry

Immunohistochemical analyses to detect PCNA, cleaved caspase-3, pIGF-1R, and pSrc expression in the xenograft and PDX tumors were performed as described previously [52]. In brief, formalin-fixed and paraffin-embedded tissue sections (4 μm) were deparaffinized, dehydrated, and treated with methanol containing 3 % hydrogen peroxide. Slides were incubated with anti-PCNA (Abcam, Cambridge, UK), anti-cleaved caspase-3 (Cell signaling), anti-pIGF-1R (Cell signaling), and anti-pSrc (Cell signaling) antibodies overnight at 4 °C, followed by incubation with biotinylated secondary antibodies (Vector Laboratories, Burlingame, CA, USA) for 1 hour at room temperature. Signals were detected using Diaminobenzidine Substrate kit (Vector Laboratories). Slides were counterstained with hematoxylin. The detailed procedures for immunohistochemical analysis and scoring of tissue microarray were described in our previous report [15].

### Statistical analysis

The data are presented as the means ± SD of a representative of at least two independently performed experiments. Statistical analysis was performed with Microsoft Excel software (Microsoft Corp., Redmond, MA, USA) or GraphPad Prism software (GraphPad Software, Inc., La Jolla, CA, USA). The quantitative analysis of blots using densitometry was performed with Image J software (National Institute of Health, Bethesda, MD, USA) [53]. The statistical significance of the data obtained from the *in vitro* experiments was analyzed using two-sided Student’s *t*-tests. The statistical significance of data obtained from the *in vivo* experiments was analyzed *via* one-way or two-way ANOVA. *P* values of 0.05 or less were considered to be statistically significant.

## Additional files

Additional file 1: Figure S1. The correlation of pIGF-1R/IR and IGF2 in human NSCLC tissue microarrays. Additional file 2: Figure S2. The correlation of pIGF-1R and pSrc in human NSCLC tissue microarrays. Assessment of significance in correlation between membrane pIGF-1R/IR and pSrc protein levels was performed using the Spearman Rank correlation test. Additional file 3: Table S1. Mutation status of NSCLC cell lines used in this study. Additional file 4: Figure S3. The expression of total and phosphorylated IGF-1R and Src in a panel of NSCLC cell lines. The protein expression was determined by Western blot analysis. Additional file 5: Figure S4. Blockade of EGF-induced IGF-1R transactivation by treatment with erlotinib but not linsitinib. Cells were pretreated with linsitinib (Linsi; 2 μM) or erlotinib (Erlo; 5 μM) for 6 h and subsequently stimulated with EGF (50 ng/ml) for 5 min. The expression of the indicated proteins was determined *via* Western blot analysis. Additional file 6: Figure S5. Densitometric quantitative analysis of pIGF-1R blots in Fig. 2e (A) and of pSrc blots in Fig. 2e (B), 2H (C), 2I (D), 2J (E), and 2K (F). Additional file 7: Figure S6. Densitometric quantitative analysis of pSrc blots in Fig. 1c. Additional file 8: Figure S7. Blockade of IGF-1R phosphorylation by treatment with increasing concentrations of linsitinib in NSCLC cells. A549 and H1975 cells were treated with increasing concentrations of linsitinib for 6 hours. The expression of the indicated proteins was determined *via* Western blot analysis. Additional file 9: Figure S8. Densitometric quantitative analysis of pSrc blots in Fig. 3a (A) and of pIGF-1R blots in Fig. 3e (B). Additional file 10: Figure S9. The effects of linsitinib on the phosphorylation of EGFR (at tyrosines 1068 and 845) and ERK in H1975 cells. H1975 cells were treated with linsitinib (2 μM) for various time points. The expression levels of pEGFR, EGFR, pERK, and ERK were determined by Western blot analysis. Additional file 11: Figure S10. The comparison of antiproliferative effects of linsitinib (1.25, 2.5, 5, and 10 μM) on the viability of low-pSrc-expressing and high-pSrc-expressing NSCLC cells. **P* < 0.05. Additional file 12: Figure S11. A time-dependent increases in total and phosphorylated IGF-1R and Src by treatment with linisitinib and blockade of these expressions by combined treatment with dasatinib. Calu-1 cells were treated with linsitinib (1 μM) for various time intervals either alone or in combination with dasatinib (100 nM) for the last 1 day. The expression levels of the indicated proteins were determined by Western blot analysis. Additional file 13: Figure S12. Blockade of linsitinib-induced IGF-1R, Src, and Akt phosphorylation by treatment with PP2 in combination. H460 cells were treated with linsitinib (1 μM) for 5 days either alone or in combination with PP2 (10 μM) for the last 1 day. The expression levels of the indicated proteins were determined by Western blot analysis. Additional file 14: Figure S13. Increases in total and phosphorylated Src and total EGFR expression by treatment with erlotinib. A549 cells were treated with indicated concentrations of erlotinib for 3 days. The expression levels of total and phosphorylated EGFR and Src were determined by Western blot analysis. Additional file 15: Figure S14. Enhanced inhibitory effects of linsitinib on the viability, anchorage-dependent colony formation, and apoptosis induction in low serum conditions. Indicated NSCLC cells were treated with the specified concentrations of linsitinib (Linsi) in media with low (1 %) or high (10 %) levels of serum for 3 days. (A) Cell viability was determined by the MTT assay. . Each bar represents the mean ± SD of a single representative experiment. **P* < 0.05, ***P* < 0.01, and ****P* < 0.001. (B) A549, H226B, and H1975 cells were treated with linsitinib (2 or 5 μM) diluted in media with low (1 %) or high (10 %) levels of serum for 12 ~ 14 days. Anchorage-dependent colony formation was determined as described in Supplemental Materials and Methods. Each bar represents the mean ± SD of at least three identical wells of a single representative experiment. ***P* < 0.01 and ****P* < 0.001. (C) *Top*. Cells were fixed with methanol and stained with PI in the presence of RNase A. Cell cycle distribution was analyzed by flow cytometry. *Bottom*. The level of cleaved PARP was determined *via* Western blot analysis. Additional file 16: Figure S15. Apoptotic cell death induced by co-targeting IGF-1R and Src. A549 and H1975 cells were treated with linsitinib alone or in combination with dasatinib for 3 days. The changes in cell cycle distribution and apoptotic cell death was determined after staining the cells with PI in the presence of RNase A by flow cytometry. Additional file 17: Figure S16. The inhibitory effect of AG1024, an IGF-1R TKI, on the proliferation of H1299 and H226Br cells. H1299 and H226Br cells were treated with AG1024 diluted in media containing low (1 %) - or high (10 %) – level of serum concentration for 3 days. Cell viability was determined by the MTT assay. Each bar represents the mean ± SD of six identical wells of a single representative experiment. Additional file 18: Figure S17. Targeting Src by PP2 treatment overcomes IGF-1R TKI resistance *in vitro* in both in high-pSrc-expressing and low-pSrc-expressing NSCLC cells. The effects on cell viability (A), anchorage-dependent (B and D) and anchorage-independent (C) colony formation were determined. The bars represent the means ± SD of a single representative experiment. **P* < 0.05, ***P* < 0.01, and ****P* < 0.001.

## Footnotes

1 Comprehensive Cancer Information, National Cancer Institute, http://www.cancer.gov/cancertopics/pdq/treatment/non-small-cell-lung/healthprofessional/page1, Accessed on September 16, 2014

2 ClinicalTrials.gov identifiers NCT01016860 and NCT01205685

3 ClinicaTlrials.gov, the U.S. National Institutes of Health, http://clinicaltrials.gov/ct2/results?term=linsitinib&Search=Search, Accessed on October 7, 2014
