# Supplementary figures and images for: Targeting the insulin-like growth factor receptor and Src signaling network for the treatment of non-small cell lung cancer
Source: Mol Cancer. 2015 Jun 4;14:113. doi: 10.1186/s12943-015-0392-3 (PMC4453276; doi:10.1186/s12943-015-0392-3)

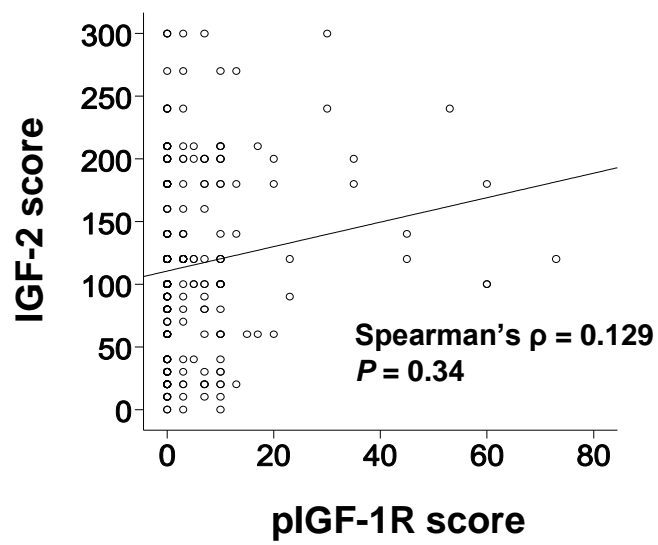

Supplement: Additional file 1: Figure S1. — The correlation of pIGF-1R/IR and IGF2 in human NSCLC tissue microarrays. [file 12943_2015_392_MOESM1_ESM.pdf]

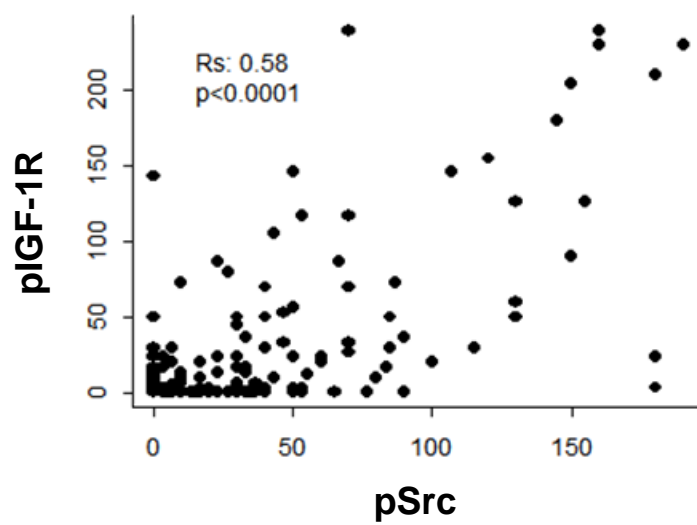

Supplement: Additional file 2: Figure S2. — The correlation of pIGF-1R and pSrc in human NSCLC tissue microarrays. Assessment of significance in correlation between membrane pIGF-1R/IR and pSrc protein levels was performed using the Spearman Rank correlation test. [file 12943_2015_392_MOESM2_ESM.pdf]

**Figure S3**

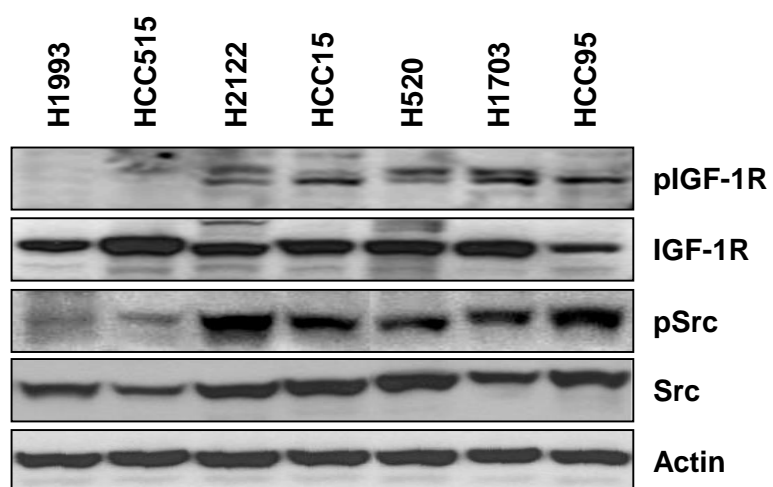

Supplement: Additional file 4: Figure S3. — The expression of total and phosphorylated IGF-1R and Src in a panel of NSCLC cell lines. The protein expression was determined by Western blot analysis. [file 12943_2015_392_MOESM4_ESM.pdf]

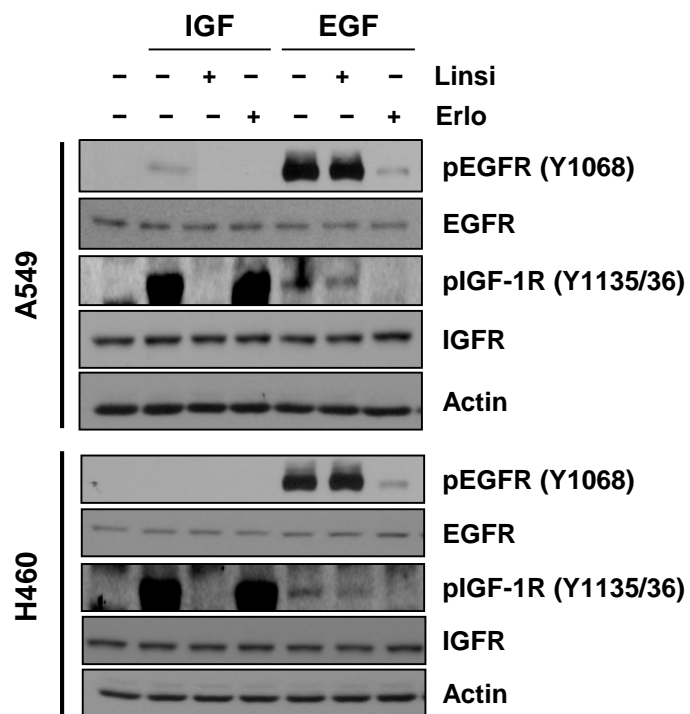

Supplement: Additional file 5: Figure S4. — Blockade of EGF-induced IGF-1R transactivation by treatment with erlotinib but not linsitinib. Cells were pretreated with linsitinib (Linsi; 2 μM) or erlotinib (Erlo; 5 μM) for 6 h and subsequently stimulated with EGF (50 ng/ml) for 5 min. The expression of the indicated proteins was determined via Western blot analysis. [file 12943_2015_392_MOESM5_ESM.pdf]

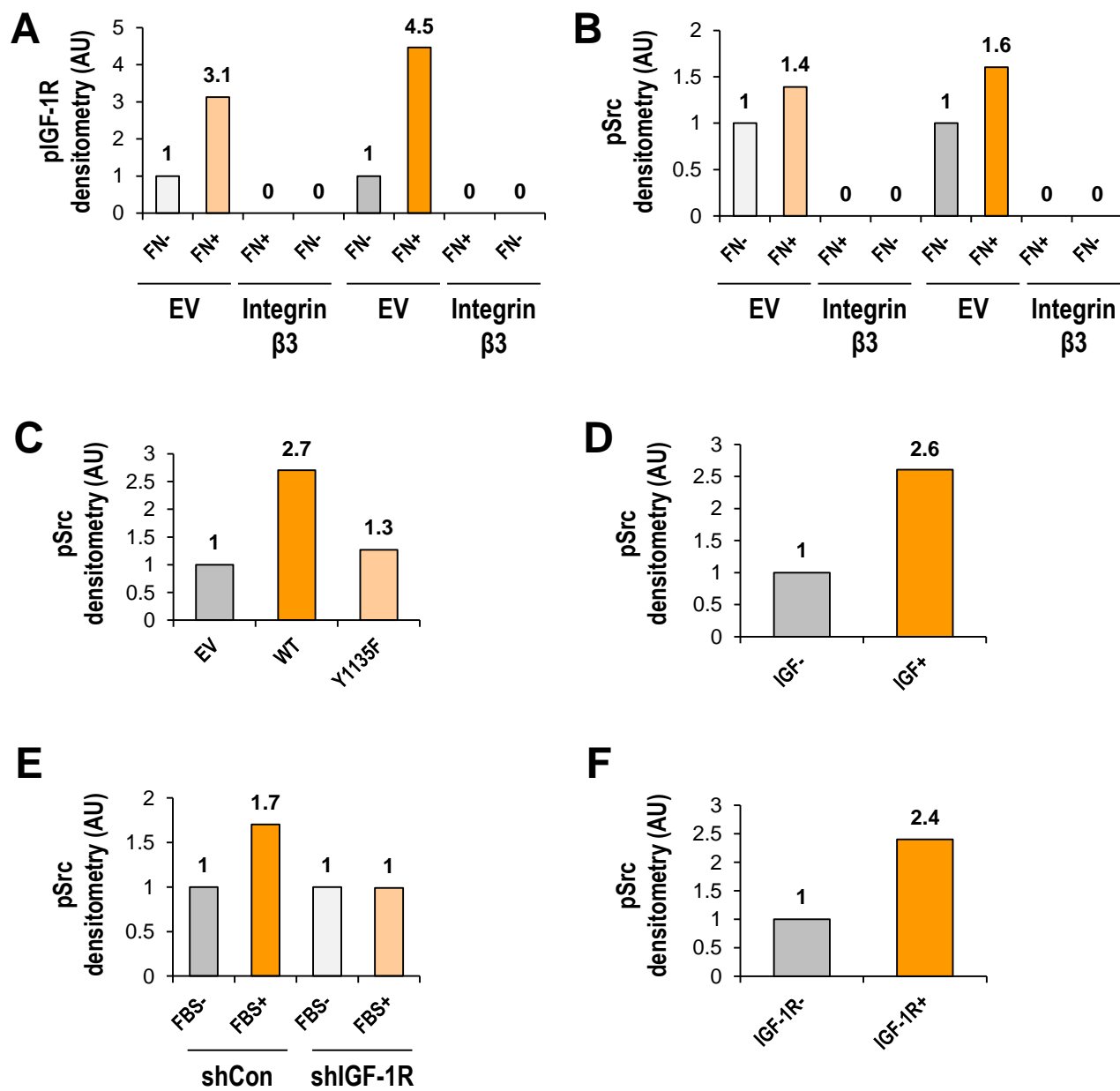

Supplement: Additional file 6: Figure S5. — Densitometric quantitative analysis of pIGF-1R blots in Fig. 2e (A) and of pSrc blots in Fig. 2e (B), 2H (C), 2I (D), 2J (E), and 2K (F). [file 12943_2015_392_MOESM6_ESM.pdf]

**Figure S6**

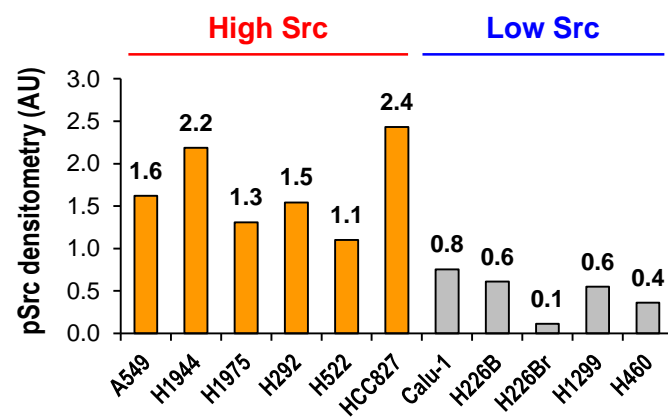

Supplement: Additional file 7: Figure S6. — Densitometric quantitative analysis of pSrc blots in Fig. 1c. [file 12943_2015_392_MOESM7_ESM.pdf]

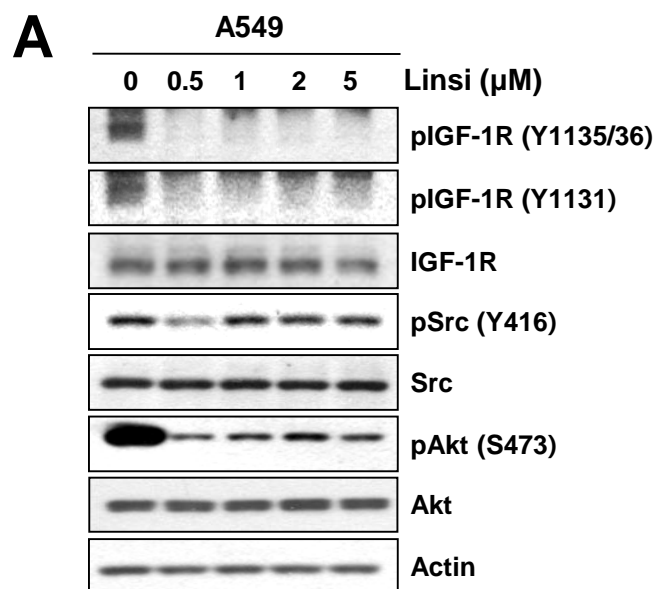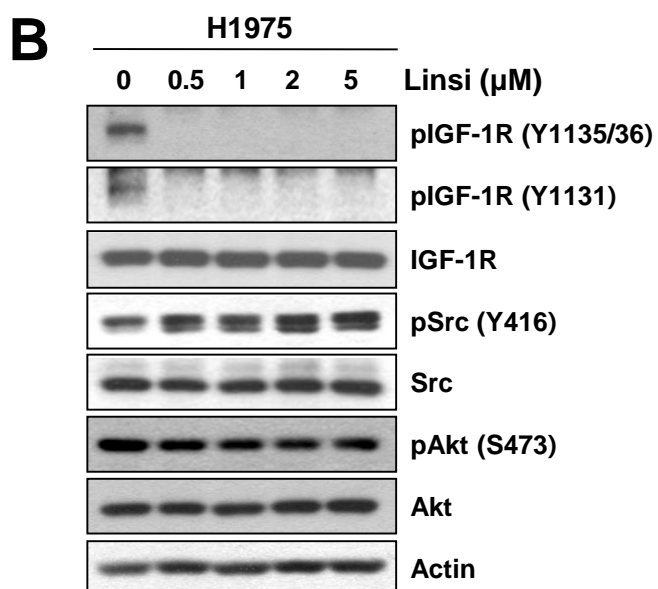

Supplement: Additional file 8: Figure S7. — Blockade of IGF-1R phosphorylation by treatment with increasing concentrations of linsitinib in NSCLC cells. A549 and H1975 cells were treated with increasing concentrations of linsitinib for 6 hours. The expression of the indicated proteins was determined via Western blot analysis. [file 12943_2015_392_MOESM8_ESM.pdf]

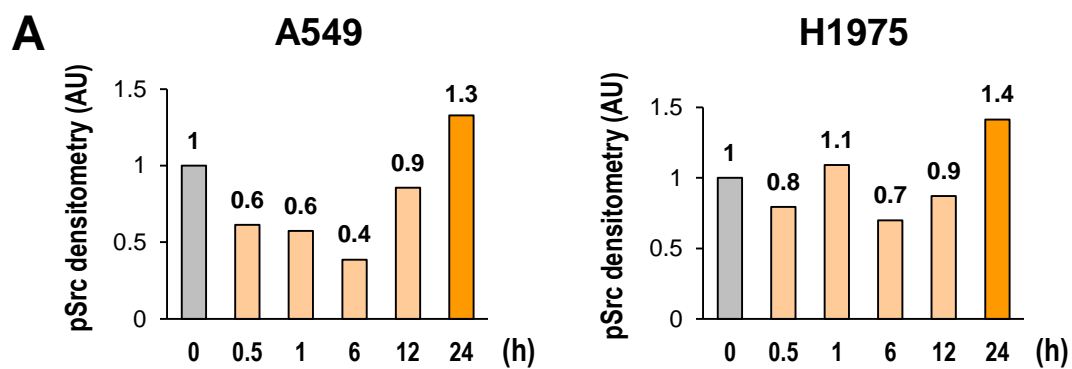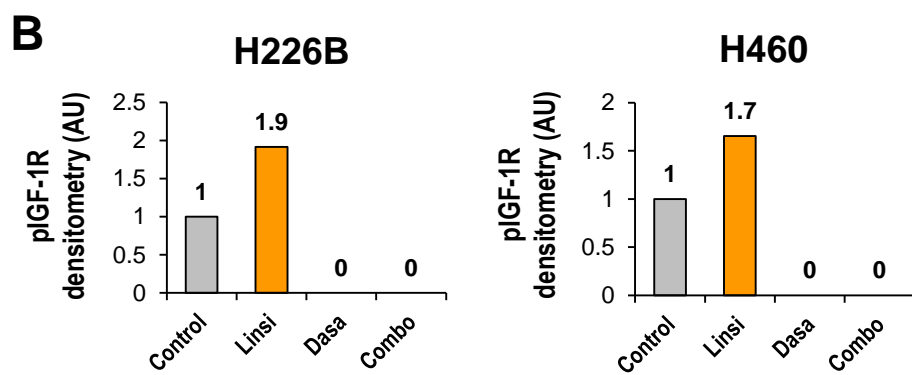

Supplement: Additional file 9: Figure S8. — Densitometric quantitative analysis of pSrc blots in Fig. 3a (A) and of pIGF-1R blots in Fig. 3e (B). [file 12943_2015_392_MOESM9_ESM.pdf]

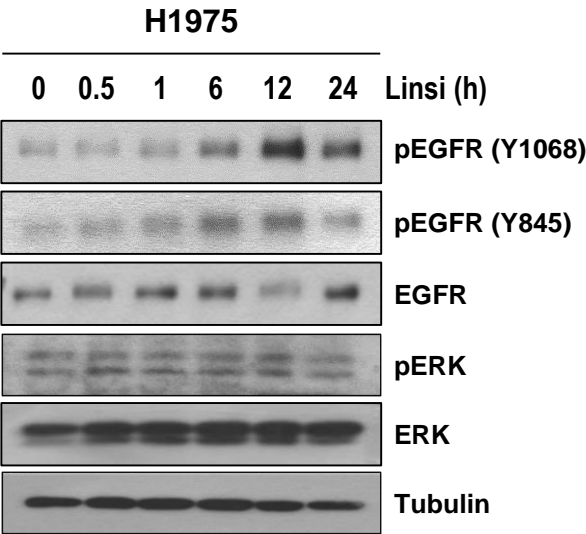

Supplement: Additional file 10: Figure S9. — The effects of linsitinib on the phosphorylation of EGFR (at tyrosines 1068 and 845) and ERK in H1975 cells. H1975 cells were treated with linsitinib (2 μM) for various time points. The expression levels of pEGFR, EGFR, pERK, and ERK were determined by Western blot analysis. [file 12943_2015_392_MOESM10_ESM.pdf]

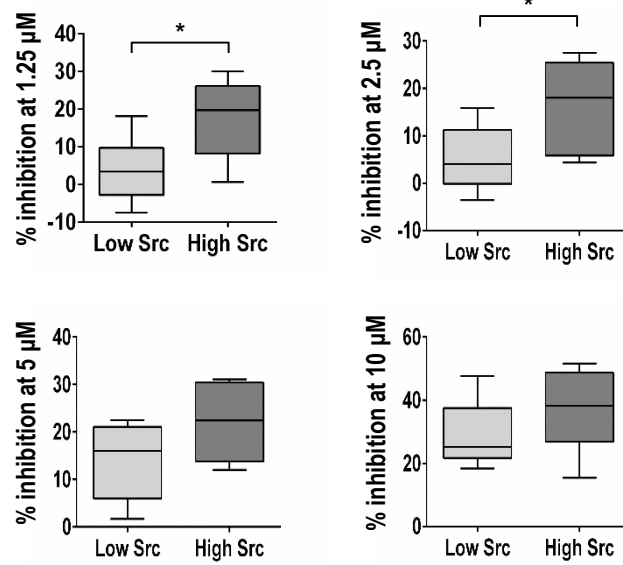

Supplement: Additional file 11: Figure S10. — The comparison of antiproliferative effects of linsitinib (1.25, 2.5, 5, and 10 μM) on the viability of low-pSrc-expressing and high-pSrc-expressing NSCLC cells. *P < 0.05. [file 12943_2015_392_MOESM11_ESM.pdf]

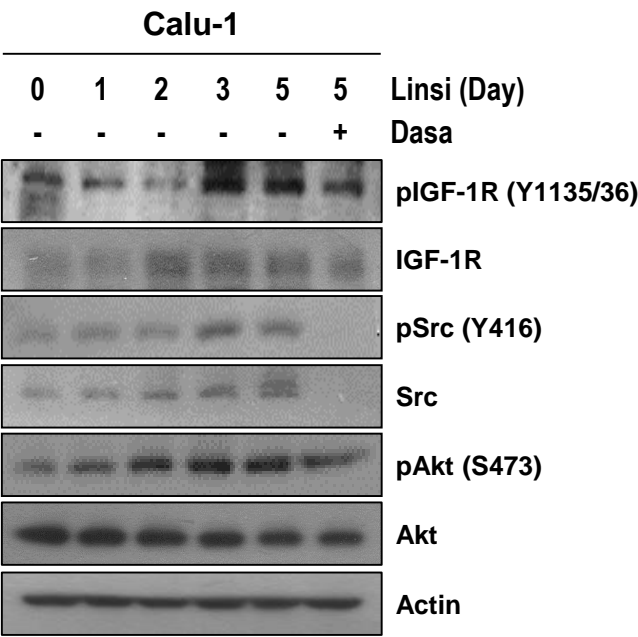

Supplement: Additional file 12: Figure S11. — A time-dependent increases in total and phosphorylated IGF-1R and Src by treatment with linisitinib and blockade of these expressions by combined treatment with dasatinib. Calu-1 cells were treated with linsitinib (1 μM) for various time intervals either alone or in combination with dasatinib (100 nM) for the last 1 day. The expression levels of the indicated proteins were determined by Western blot analysis. [file 12943_2015_392_MOESM12_ESM.pdf]

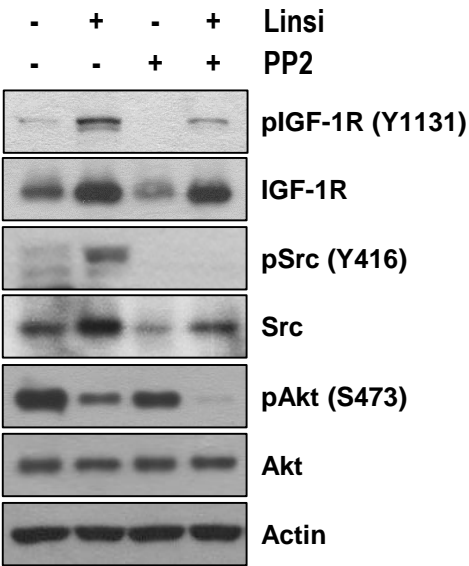

Supplement: Additional file 13: Figure S12. — Blockade of linsitinib-induced IGF-1R, Src, and Akt phosphorylation by treatment with PP2 in combination. H460 cells were treated with linsitinib (1 μM) for 5 days either alone or in combination with PP2 (10 μM) for the last 1 day. The expression levels of the indicated proteins were determined by Western blot analysis. [file 12943_2015_392_MOESM13_ESM.pdf]

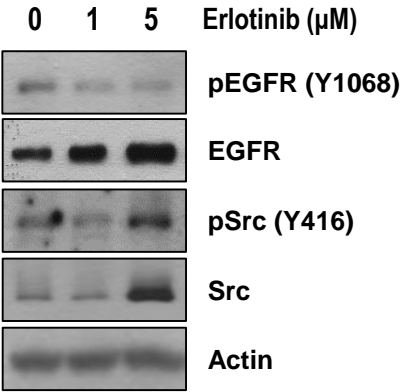

Supplement: Additional file 14: Figure S13. — Increases in total and phosphorylated Src and total EGFR expression by treatment with erlotinib. A549 cells were treated with indicated concentrations of erlotinib for 3 days. The expression levels of total and phosphorylated EGFR and Src were determined by Western blot analysis. [file 12943_2015_392_MOESM14_ESM.pdf]

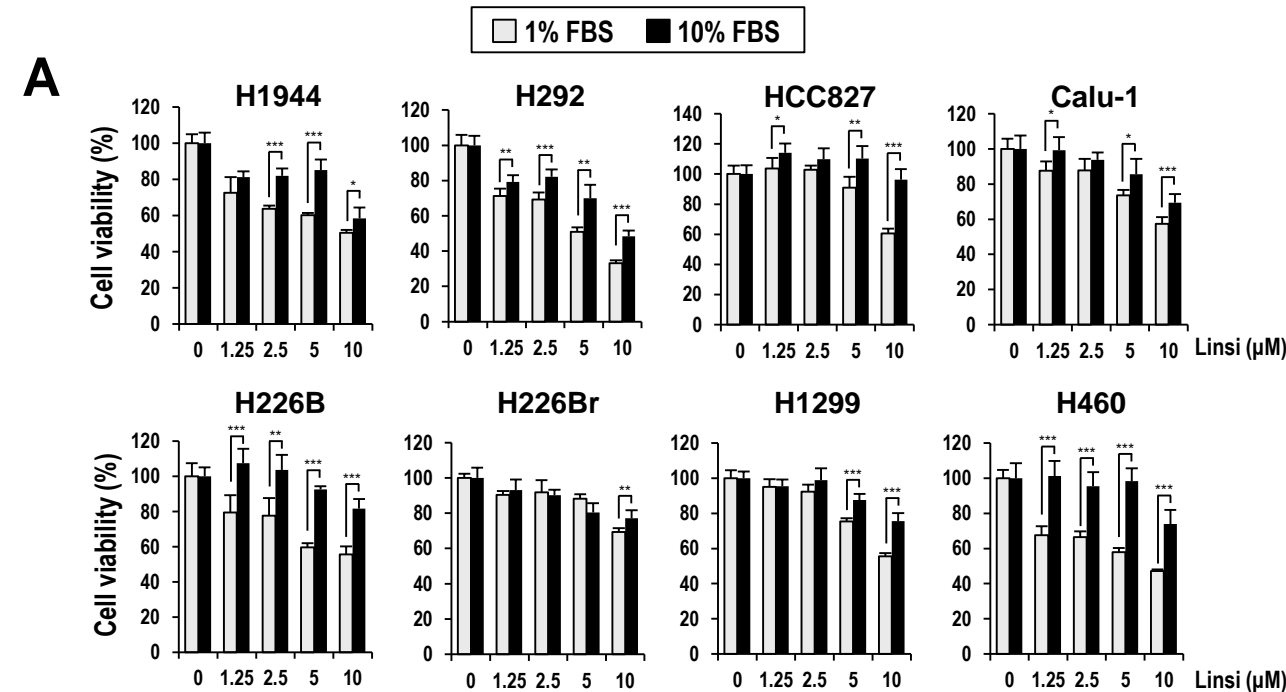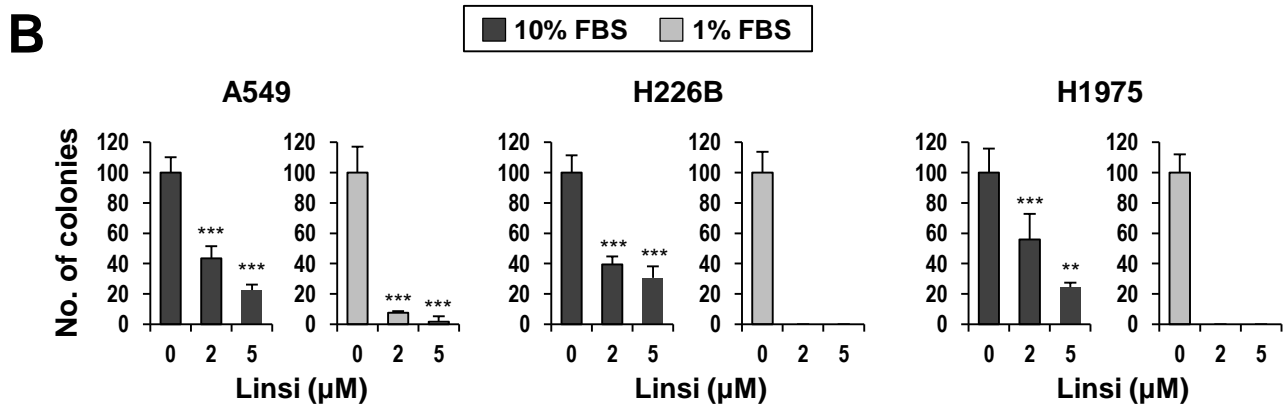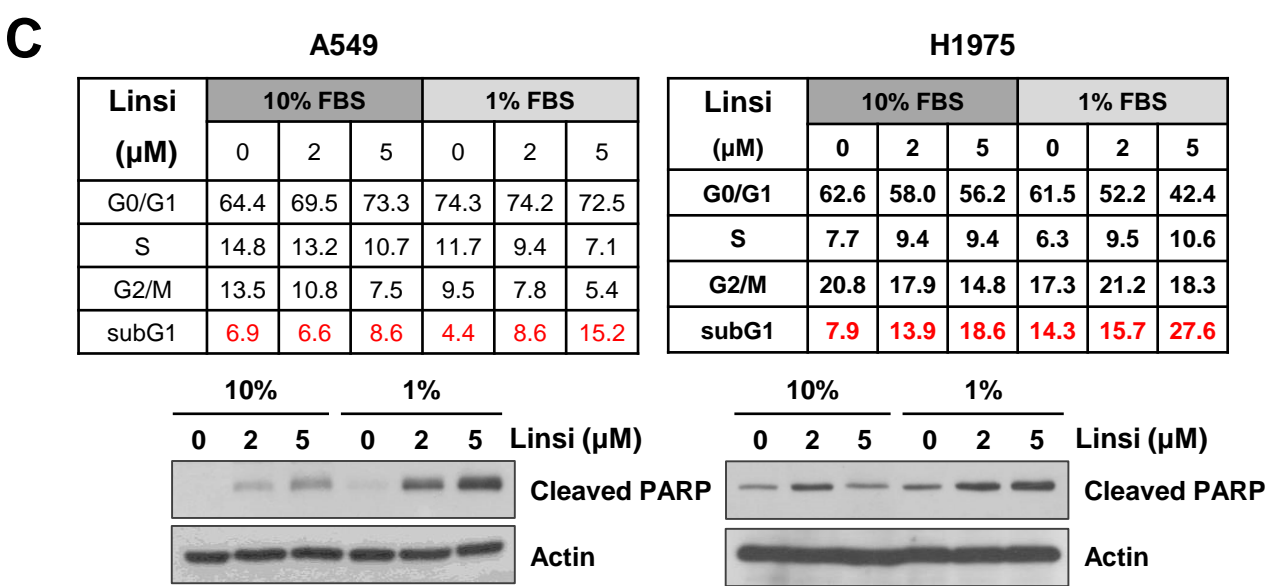

Supplement: Additional file 15: Figure S14. — Enhanced inhibitory effects of linsitinib on the viability, anchorage-dependent colony formation, and apoptosis induction in low serum conditions. Indicated NSCLC cells were treated with the specified concentrations of linsitinib (Linsi) in media with low (1 %) or high (10 %) levels of serum for 3 days. (A) Cell viability was determined by the MTT assay. . Each bar represents the mean ± SD of a single representative experiment. *P < 0.05, **P < 0.01, and ***P < 0.001. (B) A549, H226B, and H1975 cells were treated with linsitinib (2 or 5 μM) diluted in media with low (1 %) or high (10 %) levels of serum for 12 ~ 14 days. Anchorage-dependent colony formation was determined as described in Supplemental Materials and Methods. Each bar represents the mean ± SD of at least three identical wells of a single representative experiment. **P < 0.01 and ***P < 0.001. (C) Top. Cells were fixed with methanol and stained with PI in the presence of RNase A. Cell cycle distribution was analyzed by flow cytometry. Bottom. The level of cleaved PARP was determined via Western blot analysis. [file 12943_2015_392_MOESM15_ESM.pdf]

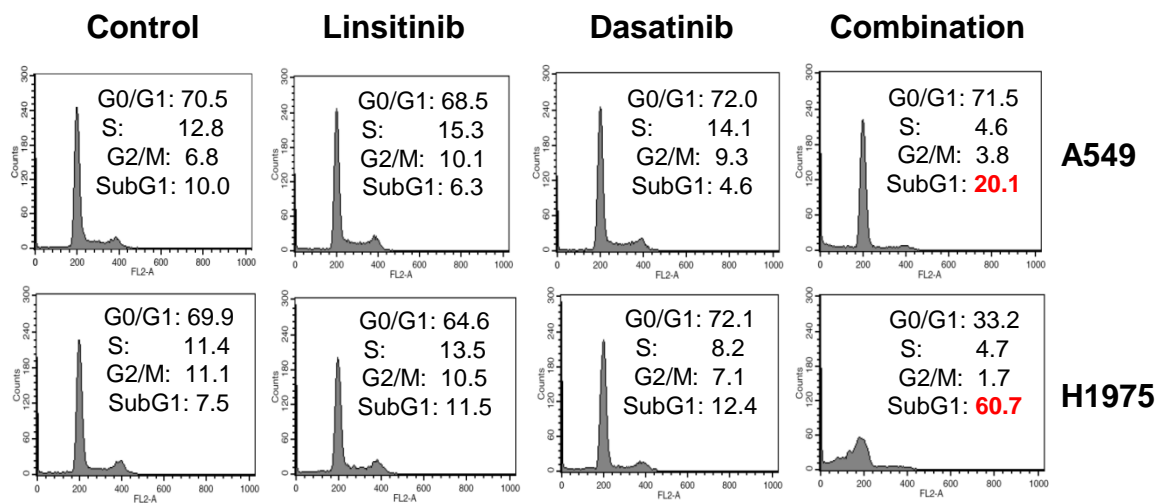

Supplement: Additional file 16: Figure S15. — Apoptotic cell death induced by co-targeting IGF-1R and Src. A549 and H1975 cells were treated with linsitinib alone or in combination with dasatinib for 3 days. The changes in cell cycle distribution and apoptotic cell death was determined after staining the cells with PI in the presence of RNase A by flow cytometry. [file 12943_2015_392_MOESM16_ESM.pdf]

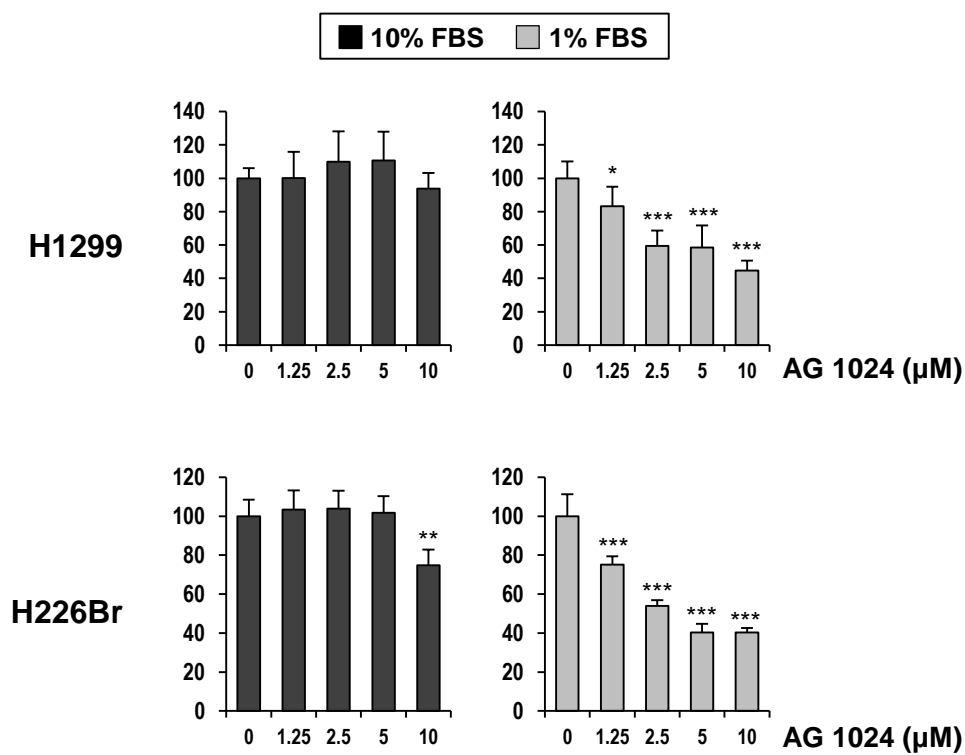

Supplement: Additional file 17: Figure S16. — The inhibitory effect of AG1024, an IGF-1R TKI, on the proliferation of H1299 and H226Br cells. H1299 and H226Br cells were treated with AG1024 diluted in media containing low (1 %) - or high (10 %) – level of serum concentration for 3 days. Cell viability was determined by the MTT assay. Each bar represents the mean ± SD of six identical wells of a single representative experiment. [file 12943_2015_392_MOESM17_ESM.pdf]

■ Linsi    ■ Linsi + PP2 (10  $\mu$ M)

**A**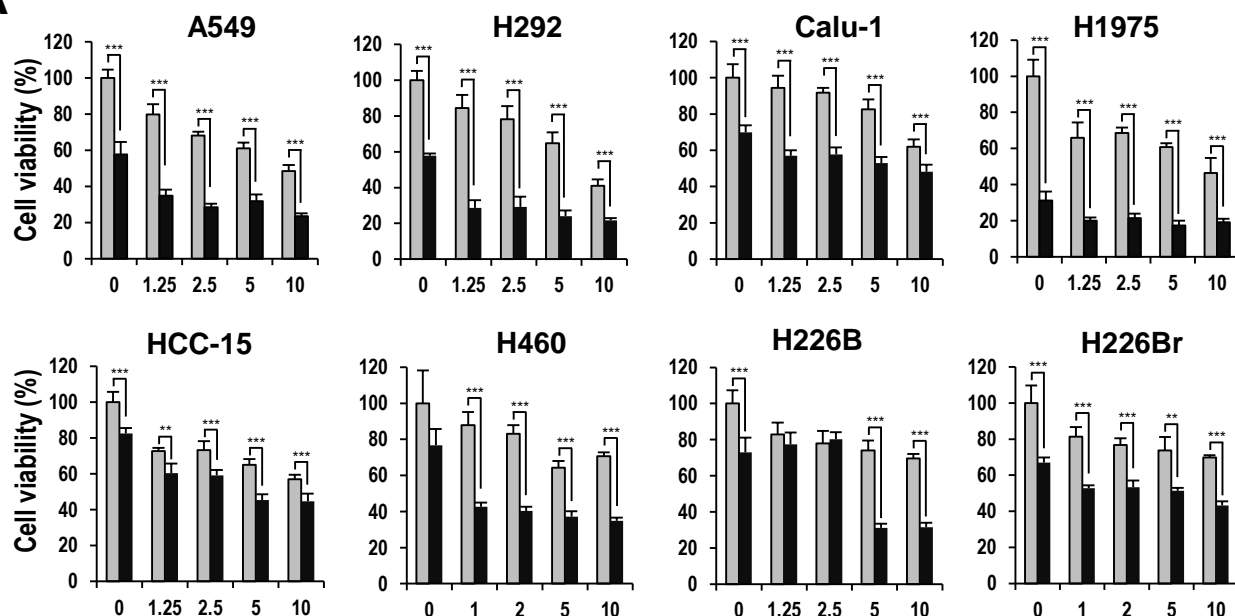**B**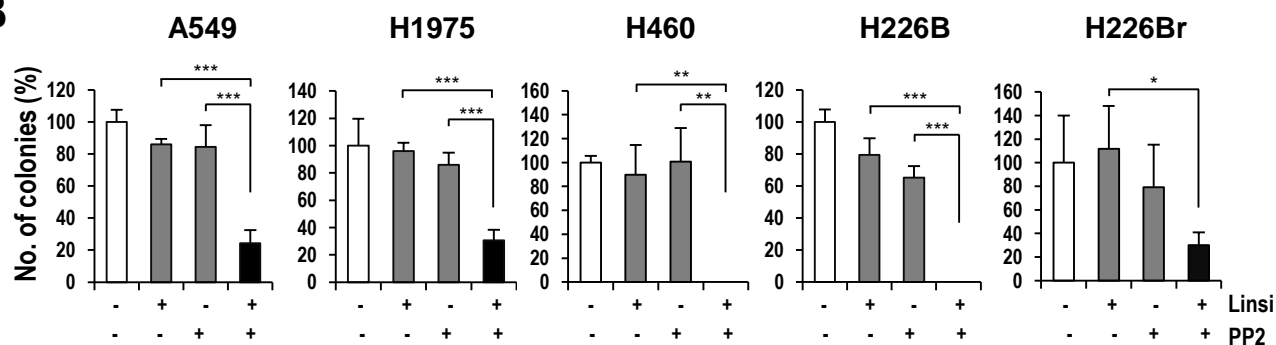**C**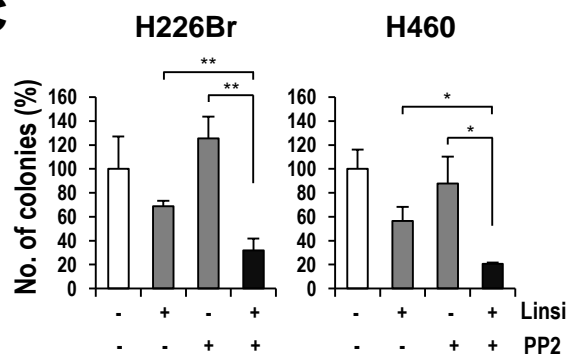**D**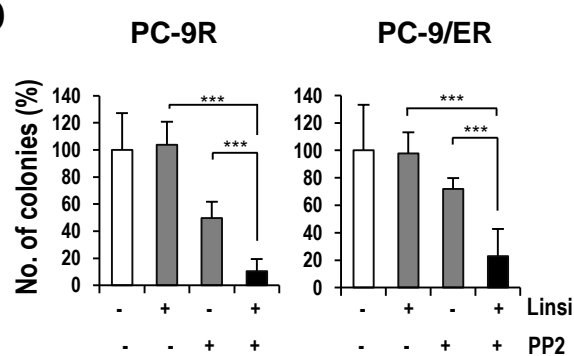

Supplement: Additional file 18: Figure S17. — Targeting Src by PP2 treatment overcomes IGF-1R TKI resistance in vitro in both in high-pSrc-expressing and low-pSrc-expressing NSCLC cells. The effects on cell viability (A), anchorage-dependent (B and D) and anchorage-independent (C) colony formation were determined. The bars represent the means ± SD of a single representative experiment. *P < 0.05, **P < 0.01, and ***P < 0.001. [file 12943_2015_392_MOESM18_ESM.pdf]
