# Supplementary material for: Targeting the insulin-like growth factor receptor and Src signaling network for the treatment of non-small cell lung cancer
Source: Mol Cancer. 2015 Jun 4;14:113. doi: 10.1186/s12943-015-0392-3 (PMC4453276; doi:10.1186/s12943-015-0392-3)
Supplement: Additional file 3: Table S1. — Mutation status of NSCLC cell lines used in this study. [file 12943_2015_392_MOESM3_ESM.pdf]

Table S1

	Adenocarcinoma						Squamous cell carcinoma					Large cell carcinoma	
	A549	H1944	H1975	H292	H522	HCC827	Calu-1	H226B	H226Br	H520	HCC15	H1299	H460
EGFR	WT	WT	Mut	WT	WT	Mut	WT	WT	WT	UD	WT	WT	WT
K-Ras	Mut	Mut	WT	WT	WT	WT	Mut	WT	Mut	WT	WT	WT	Mut
p53	WT	WT	Mut	WT	Mut	WT	WT	WT	Mut	Mut	Mut	Nu	WT
PTEN	WT	U	WT	WT	WT	WT	WT	U	U	WT	WT	Nu	WT
PI3KCA	WT	U	Mut	WT	WT	Mut	WT	WT	U	WT	WT	WT	Mut
LKB1	Mut	WT	WT	WT	WT	WT	WT	WT	U	WT	Mut	WT	Mut
B-Raf	WT	U	WT	WT	WT	U	WT	WT	U	WT	WT	WT	WT

WT: wild type Mut: mutant type Nu: null U: unknown UD: undetectable
